# Gene expression adaptation of metastases to their host tissue

**DOI:** 10.1016/j.isci.2026.116744

**Published:** 2026-07-21

**Authors:** Luise Nagel, Marten Wenzel, Sascha Hoppe, Mohammad Karimpour, Patrick S. Plum, Abdossamad Hamoudi, Israt Jahan, Sarah Schmitt, Marek Franitza, Roger Wahba, Marc Bludau, Christiane J. Bruns, Alexander Quaas, Andreas Beyer, Axel M. Hillmer

**Affiliations:** 1 University of Cologne, Faculty of Mathematics and Natural Sciences, Cologne Excellence Cluster for Aging and Aging-Associated Diseases (CECAD), Cologne, Germany; 2 Charité-Universitätsmedizin Berlin, Department of Biochemistry, Berlin, Germany; 3 University of Cologne, Faculty of Medicine and University Hospital Cologne, Institute of Pathology, Cologne, Germany; 4 University of Cologne, Faculty of Medicine and University Hospital Cologne, Department of General, Visceral, Cancer and Transplantation Surgery, Cologne, Germany; 5 Department of General, Visceral and Transplant Surgery, University Hospital Tübingen, Tübingen, Germany; 6 University of Cologne, Cologne Center for Genomics, Cologne, Germany; 7 Department of General, Visceral and Oncologic Surgery, Helios Klinikum Berlin-Buch, Berlin, Germany; 8 University of Cologne, Center for Molecular Medicine Cologne, Cologne, Germany

**Keywords:** colorectal cancer, liver metastasis, single cell sequencing, adaptation

## Abstract

Adaptation of metastatic cells to their host tissue determines the pathogenicity of cancer. Yet, it remains elusive to what extent the host environment drives gene expression programs in metastatic cells. We present a new concept to identify adaptive mechanisms that enable metastases to establish themselves in a novel tissue context. We generated quadruple-paired single-cell RNA-sequencing data from malignant and benign tissues from untreated donors with colorectal adenocarcinoma and liver metastasis. Utilizing a computational approach, we deduce tissue-adaptive expression patterns by identifying genes that consistently adapt to the host tissue. Expression changes in metastases are reminiscent of benign liver epithelial cells, including basic cellular functions such as energy metabolism, as well as tissue-specific pathways such as the regulation of lipid metabolism by peroxisome proliferator-activated receptor alpha (PPAR-α). This approach identifies the molecular adaptation of cancers to the host environment, which potentially increases the pathogenicity of metastatic cells, proposing a new therapeutic strategy to target adaptive processes.

## Highlights

• A two-dimensional expression analysis allows the identification of metastatic adaptation
• Liver metastases of colorectal cancer change their transcriptomes to a liver-like phenotype
• Transcriptomic changes in liver metastases reflect metabolic rewiring
• PPAR-*α* signalling is up and oxidative phosphorylation down-regulated in liver metastases

## Graphical abstract

**Figure:**
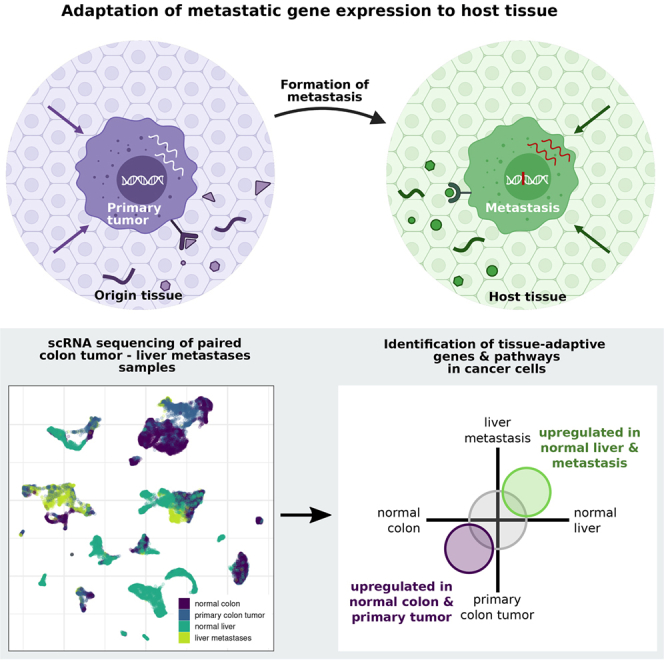

## Teaser

Oncology; Precision medicine; Computational bioinformatics; Systems biology; Cancer systems biology; Omics

## Introduction

The formation of distant metastases necessitates cancer cells to leave the primary cancer site, travel as a circulating tumor cell to another organ, infiltrate this organ, and adapt to the novel surrounding tissue and grow as a metastasis (MT).^1^^,^^2^^,^^3^ These obstacles result in less than 0.01% of the primary tumor (PT) cells metastasizing.^4^ The fact that a significant number of cancer cells can circulate in the bloodstream but form a limited number of metastases, known as the ineffectiveness of metastatic spread,^5^^,^^6^^,^^7^^,^^8^ illustrates the barrier cancer cells have to overcome to survive and proliferate in a novel tissue context. Given that metastases are responsible for over 90% of cancer-related deaths, enhancing our understanding of this process is of great importance to increase patient survival.^9^ In the 19th century, Stephen Paget suggested the “seed and soil” theory, saying that a cancer cell (seed) requires a specific tissue (soil) to form a MT.^10^ The role of the PT features for the formation of metastases has recently been described for colorectal cancer (CRC). Here, larger primary CRCs give rise to metastases with a non-desmoplastic growth pattern, while CRCs with high-grade inflammation lead to liver metastases with desmoplastic histology. It was also noted that lymphovascular invasion is associated with fewer but earlier and metachronous liver metastases.^11^ Further, it was recognized that patterns of metastases are influenced by the architecture of tissues and the bloodstream.^12^ While both factors are valid and contribute to the likelihood that a metastasis forms, the ability of a cancer cell to adapt is an additional pivotal factor for the establishment of metastases.

The specific gene expression signature of metastatic cells is determined by four factors: (1) the tissue of origin,^13^ (2) the cancer-specific expression signatures,^14^^,^^15^^,^^16^^,^^17^^,^^18^ (3) the metastatic process, and (4) adaptation to the host tissue. Expression patterns of metastases show a strong similarity to that of the PT they originate from,^13^ through the presence of two of the four key characteristics listed above: (1) resemblance to the expression signature of the original cell type from which the tumor arose and (2) cancer-specific alterations. Moreover, MT-specific processes such as epithelial-mesenchymal transition (EMT) and mesenchymal-epithelial transition (MET), including dissemination, travel, invasion,^1^^,^^15^^,^^19^^,^^20^^,^^21^^,^^22^ as well as escaping the immune system,^23^^,^^24^^,^^25^ necessitate the involvement of specific pathways and functions, resulting in an (3) altered gene expression pattern that is distinct from that of the PT.^1^^,^^19^ Furthermore, the expression profile of the metastases is driven by (4) the novel surrounding tissue in which they establish themselves, as cancer cells undergo a tissue-specific metabolic adaptation process to integrate and survive in a new organ.^26^ Metastases need to display a high metabolic flexibility, enabling them to accommodate different environments with different oxygen abundances, energy sources, and nutrient availability.^27^^,^^28^ Importantly, while this adaptation may be enhanced or “helped” by genetic and/or epigenetic predisposition in metastasizing cells, such (cell-intrinsic) predisposition is not sufficient to explain all MT-specific processes. Instead, this adaptation also results from cell-extrinsic signals in the new environment,^29^ such as local nutrient availability and paracrine signaling resulting from tissue-resident cells. In addition to explaining the establishment of metastases in their new environment, this adaptation may also modulate the pathogenicity of metastases independent of their genotype. Thus, better understanding the interaction between metastases and their environment is of utmost clinical importance.

Monitoring gene expression in metastatic cells has great potential for elucidating the adaptive processes enabling metastasizing cells to successfully establish themselves in the new tissue environment. In the case of CRC, the comparison of primary CRC with liver MT bulk transcriptomics has led to the identification of 22 liver characteristic genes that are upregulated in MT.^30^ Selected drugs targeting related pathways showed growth inhibitory activity for some patient-derived organoids,^30^ suggesting that pharmacological control of liver metastases might be feasible. Earlier work has focused on the direct comparison of gene expression differences between metastases and their PTs without addressing the question to what extent those differences may be driven by metabolic adaptation.^15^^,^^19^^,^^20^ Other research has focused on the direct interaction between the MT and its microenvironment, with a specific focus on immune cells.^23^^,^^24^^,^^25^ Although this research is highly relevant for understanding immune evasion, it does not address the question of metabolic tissue adaptation. Further, two liver-specific genes, *ANGPTL3* and *CFHR5*, were found to be expressed in CRC PTs of patients who developed liver metastases, in contrast to non-metastasizing CRC. Both genes are involved in tyrosine and drug metabolism, indicating that metabolic changes of CRC cells might be important for the formation of liver metastases.^31^ More recently, a combination of bulk transcriptomics and xenograft-based single cell sequencing approaches has been used to investigate cell type-specific changes in CRC-derived liver metastases, pointing to liver-specific transcriptional factors that become activated in CRC liver MT.^32^ Epigenetic single cell analyses of such xenograft experiments revealed cell modules in liver metastases with changes in oxidative phosphorylation and lipid metabolism.^33^ Single-cell transcriptomics identified specific T cells as increased in liver metastases and MCAM+ fibroblasts potentially influencing such cell populations.^34^ Comparing CRC/liver metastases with and without perioperative chemotherapy and a focus on the tumor microenvironment (TME) showed more activated B cells, reduced tumor-associated macrophage diversity, fewer T cells, and less extracellular matrix remodeling in cancer-associated fibroblasts in chemotherapy treated metastases.^35^ Overall, it clearly shows that liver metastases differ from primary CRC tumors with regard to their TME. However, the changes within patients’ cancer cells themselves are understudied so far. While an adaptation of breast cancer cells to lung metastases and related oxidative stresses has been demonstrated,^36^ it remains underexplored how CRC cells in patients adapt when they form liver metastases.

Here, we present a novel concept that combines experimental and bioinformatic methodology for the identification of tissue-adaptive genes and pathways, i.e., genes/pathways whose altered expression in metastases reflects an adaptation to the new host tissue. This novel concept is based on the simultaneous single-cell profiling of four cellular compartments from the same donor: primary colon tumor cells, tumor cells of the MT, and benign cells from the two respective host organs. Importantly, benign cells are sampled distant from the tumor sites to minimize the direct influence of the tumor on them. This quadruple-paired design enables us to not only identify expression differences between the MT and the PT but also to integrate these with the expression differences between the two healthy tissues to identify tissue-adaptive expression patterns that are conserved across donors (Figure S1). Having matching samples from the same donors is pivotal for the identification of tissue-adaptive expression patterns. To the best of our knowledge, this is the first instance in which a quadruple-paired design has been employed for this purpose.

Once cancer has developed metastases and turned into systemic disease, surgery with curative intent is usually not beneficial anymore, resulting in a scarcity of clinical specimens suitable for single-cell sequencing approaches. CRC that formed a limited number of distinct detectable synchronous metastases in the liver belongs to the rare clinical situations where, despite metastases, surgery increases chances for prolonged survival and therefore is used in the clinic.^37^^,^^38^ Usually, however, neoadjuvant or perioperative therapy is applied before surgery to such patients, in the context of multimodal therapeutic approaches, which may lead to persisting changes in cell biological processes in the metastases. We have deliberately selected patients without neoadjuvant treatment and simultaneous surgery on colon and liver to get an unbiased view on the cell states and respective transcriptomes of CRC cells of the PT and metastases within the same donor together with cells of the normal tissues.

As metastasizing cells have to integrate and survive in a novel tissue context, we hypothesized that liver metastases of colon cancer change their gene expression profile toward a liver-like phenotype. Using primary colon cancer with liver metastases as a model system, we analyze the adaptation of cancer cells to their novel environment. This analysis reveals that the majority of expression changes in the MT reflect an expression signature reminiscent of benign liver epithelial (LE) cells. Cellular processes adapting to the liver environment include basic cellular functions such as energy metabolism, as well as tissue-specific pathways such as the regulation of lipid metabolism by peroxisome proliferator-activated receptor alpha (PPAR-α).

## Results

### Single-cell RNA sequencing of paired colon cancers and liver metastases

**Figure 1:**
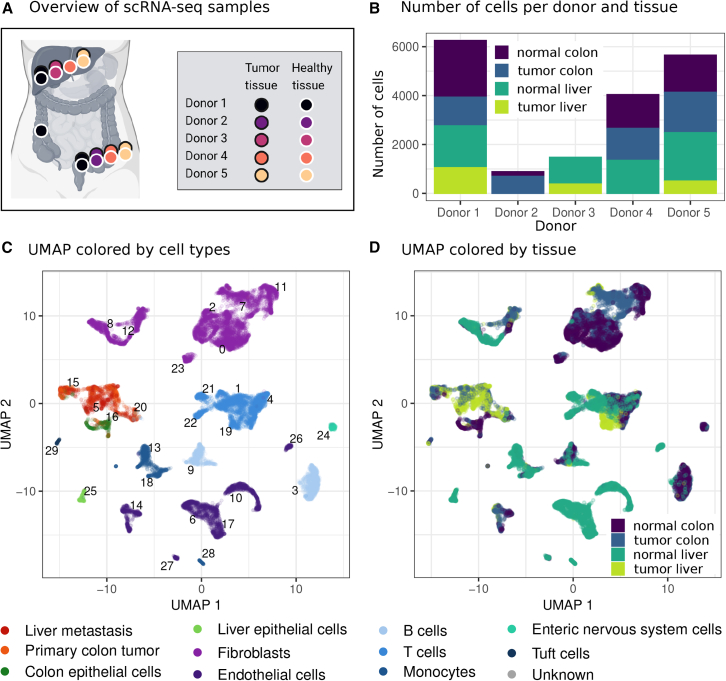
Overview of our scRNA-seq dataset from five donors with primary colon tumor and liver metastasis (A) Overview of scRNA-seq samples available from each donor (partly created with BioRender.com.). (B) Number of cells per donor colored by sample origin site (normal colon (purple), tumor colon (blue), normal liver (turquoise), and tumor liver (light green). (C) UMAP of scRNA-seq data with colors indicating different cell types. Cluster numbers are shown in the plot. (D) UMAP of scRNA-seq data with colors indicating different sample site origins. Colors as in b).

Matching tissue from five donors with untreated CRC and liver MT were sampled and underwent scRNA-seq (Figures 1A and 1B, and Table S1, see STAR Methods). We performed scRNA-seq on tumor samples (which consist of a mix of benign and tumor cells) and healthy tissue distant from the tumor. After stringent quality control, a total of 18,312 cells of 16 tissue samples with 26,807 detected genes (65% protein coding) were obtained (Figure 1C and Table S2). After normalization and integration, we grouped the cells based on their expression pattern, resulting in 30 distinct clusters (Figure S2A and Table S2). Differential expression analysis was applied to identify cluster-specific marker genes (see STAR Methods), and cell types were annotated utilizing previously known cell type-specific expression patterns (Figure 1C). Clusters commonly consisted of cells from different donors (Figures S2B–S2D) and 22 of 30 clusters were composed of cells from liver and colon (Figure 1D). The most frequent cell type found across all tissues was fibroblasts, making up the majority of cells in both the healthy and tumor colon samples.

### Differentially expressed genes between primary tumor and metastasis cells

**Figure 2:**
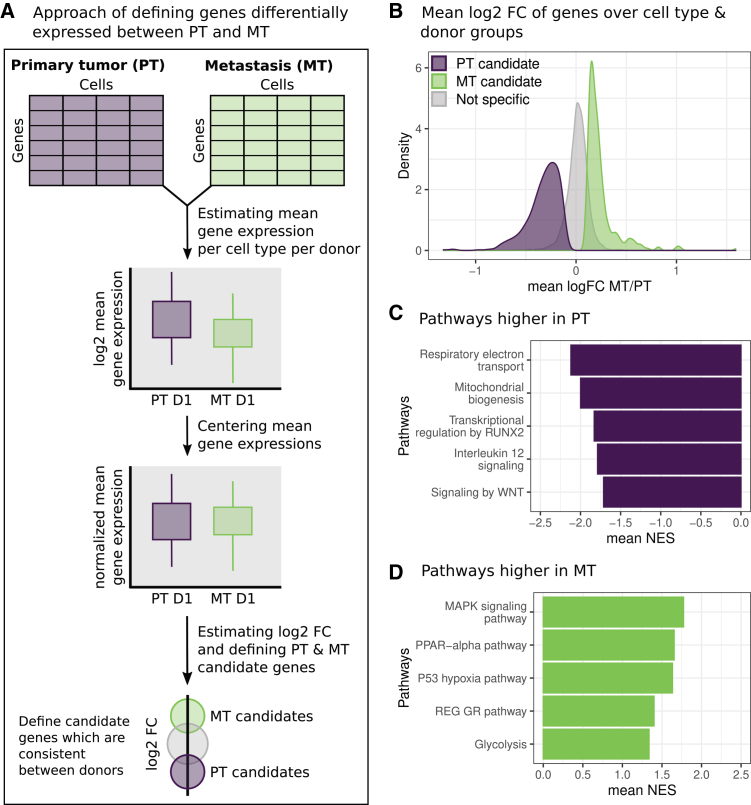
Gene expression differences between metastasis and primary tumor cells (A) Computational approach for defining differentially expressed genes between MT and PT in different donors. Exemplified here for donor 1 (D1). (B) Average log2-fold change (FC) of centered mean gene expression over donors between MT and PT. PT-specific (purple), MT-specific (green), and non-differentially expressed genes (gray) are plotted individually. (C) Five of the most upregulated pathways in PT cells when compared to MT cells. The mean normalized enrichment score (NES) across the donors is displayed. (D) Five of the most upregulated pathways in MT cells compared to PT cells. Mean NES across the donors is displayed.

For the identification of differences in gene expression between cancer cells from the PT versus cancer cells from the MT, we searched for genes that are consistently higher in the PT or MT across multiple donors. Using all tumor cells from donors with both PT and MT data available, we estimated donor-specific pseudo-bulk expression profiles and fold changes for all genes that were consistently expressed in the primary cancer and MT cells (*n* = 6,872 (98% protein coding) from 26,807 genes detected in total, see STAR Methods). Genes that were overexpressed in either the MT or the PT across all donors were defined as “metastasis specific” (MT-specific; 305/6,872) or “primary tumor specific” (PT-specific; 530/6,872 genes), respectively (Figures 2A and 2B, and Table S3, see STAR Methods). A small number of genes (131/24,642 with 24,642/26,807 genes included in the analysis as they are expressed in at least one of the cell types of interest) were found to be expressed in a tissue-exclusive manner, as they were virtually undetectable in the respective other tissue (PT exclusive genes: 49/24,642, MT exclusive genes: 82/24,642, Table S4, see STAR Methods).

Many of the genes that we identified as being more strongly expressed in the MT can be linked to the metastatic process, such as the cysteine-rich intestinal protein (*CRIP*), which is upregulated in the MT cells. *CRIP1* is known to be involved in cell migration and invasion in various cancer types, including CRC.^39^ *CRIP1* mediates the EMT and is therefore directly involved in MT formation.^40^^,^^41^ Fatty acid-binding protein 1 (*FABP1*) was consistently higher expressed in the liver MT compared to the PT. *FABP1* is involved in the binding, transport, and metabolism of long-chain fatty acids,^42^^,^^43^ and its elevated expression has been reported for various tumor types, including colorectal and hepatocellular carcinomas.^44^ Although *FABP1* has not yet been directly associated with MT formation, exogenous *FABP* expression has, however, been associated with tumor progression and invasiveness.^45^

Pathway enrichment analysis revealed differences in the energy metabolism between the PT and the MT (Figures 2C and 2D, and Table S5, see STAR Methods). While glycolysis was upregulated in the MT, genes involved in oxidative phosphorylation (respiratory electron chain and mitochondrial biogenesis) were more highly expressed in the PT. This is consistent with previous reports, showing that glycolytic dominant metabolic reprogramming commonly occurs during the metastatic process, resulting in an increase in glycolysis in the MT compared to the PT.^27^^,^^46^^,^^47^ Cells adapt their energy metabolism to the environment. For example, hypoxia is known to drive cells into glycolysis, and consistent with that notion, we observed a slight upregulation of Hypoxia Inducible Factor 1 Subunit Alpha (*HIF1A*) in metastatic cells.^48^^,^^49^

### Adaptation of gene expression to the novel tissue context

Next, we wanted to test the hypothesis that some of the gene expression changes that we observed at the transition from the PT to the MT resulted from the adoption of a “liver-like” gene expression program. One example of this is the PPAR-*α* pathway that was upregulated in the metastatic cells compared to the PT (Figure 2D). This observation could serve as a first hint toward a metabolic adaptation of metastatic cells to the liver environment, as PPAR-*α* signaling is reduced in the colon but gets activated via paracrine liver signaling and plays a key role in hepatocyte biology.^50^^,^^51^ Performing pathway enrichment on the log2 fold changes between normal liver and normal colon epithelial (CE) cells revealed organ-specific differences in pathway expression (exemplified in Figures S3A and S3B; complete list in Table S5). The PPAR-*α* pathway, which we found to be upregulated in MT compared to PT, is also higher expressed in LE cells compared to CE cells. Other examples of pathways higher expressed in LE cells include pathways such as the *HNF3A pathway*^52^ (FOXA1 transcription factor network), where FOXA1 is known to mediate liver development by regulating hepatocyte and cholangiocyte growth,^53^ or the *insulin signaling pathway*,^54^ which is expected to be higher in LE cells^55^^,^^56^ as the liver is the principal insulin-responsive metabolic organ. Similarly, pathways we found to be higher expressed in colon than in LE cells include colon epithelium specific regulations such as the *metabolism of polyamines*,^57^ which regulates epithelial proliferation in the colon,^58^ or the *propanoate metabolism*.^59^ Gut bacteria ferment carbohydrates into short-chain fatty acids such as propionate, which is subsequently absorbed by colonocytes^60^ and acts as a local energy source^60^^,^^61^ and signaling molecule.^62^

**Figure 3:**
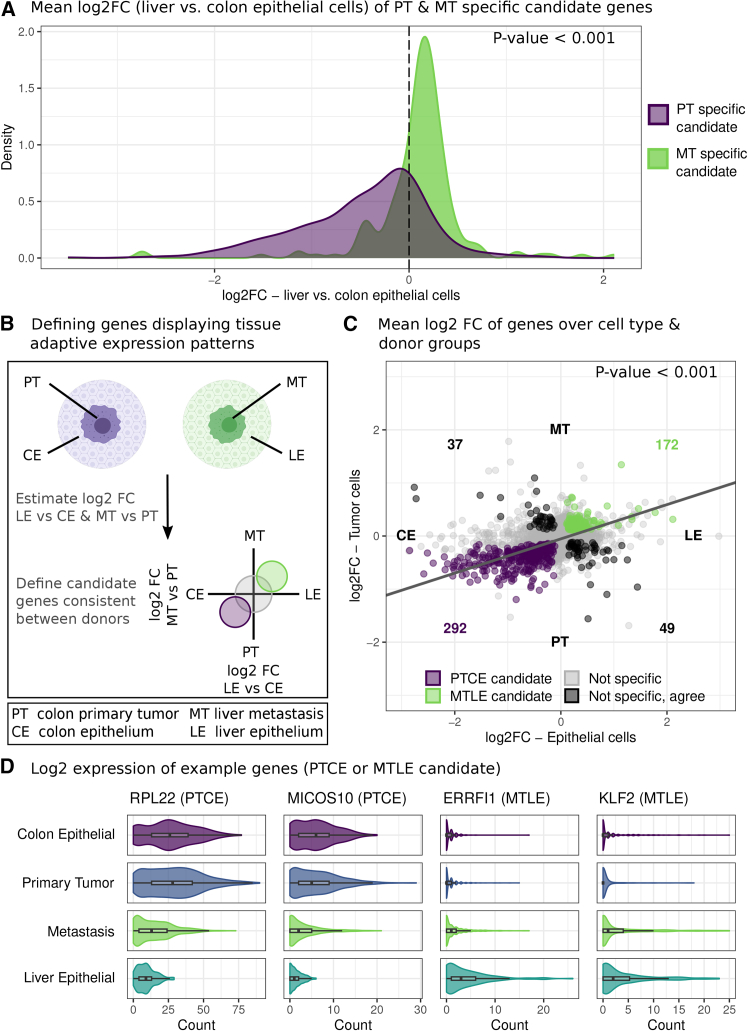
Gene expression differences between metastasis and primary tumor as well as liver and colon epithelial cells (A) Mean log2-fold change of liver to colon epithelial cells of genes defined as primary tumor (purple) or metastasis (green) specific in 3a and b. The log2 FC of the epithelial cells of the PT-specific genes significantly differs from those of the MT-specific candidate genes (Wilcoxon rank-sum test <0.001). (B) Bioinformatic pipeline for defining genes that are differentially expressed between metastasis and primary tumor as well as liver and colon epithelial cells in different donors. (C) Mean log2-fold change of metastasis and primary tumor plotted against the mean log2-fold change of liver and colon epithelial cells estimated over the donors (8 samples). Genes with expression patterns consistent between the donors are highlighted (primary tumor and colon epithelial specific genes = purple, metastasis and liver epithelial specific genes = green, other = black; four outlier genes are not visualized in the plot). The deming regression coefficient is displayed as a gray line. distribution of the PTCE & MTLE genes significantly differs from (A) a random distribution (*p* value <0.001, Pearson’s Chi-squared Test) and (B) the non-candidate genes (*p* value <0.001, Pearson’s Chi-squared Test). (D) Expression of four example candidate genes (PTCE candidates: RPL22 & MICOS10, MTLE candidates: ERRFI1, KLF2) across all cells of donors included in this analysis (8 samples). Box limits represent the interquartile range (IQR; 25th to 75th percentiles), the solid line inside the box indicates the median, and the whiskers extend to data points within (1.5 × IQR) from the quartiles. Outliers are not shown.

We expected that the expression patterns of “tissue-adaptive genes” would agree between metastatic colon cancer and normal liver cells, as both reside in the same environment. To test this hypothesis, we focused on epithelial cells, because the PTs arose from CE cells, and epithelial cells are the main cell type present in the liver, where MT manifested. When comparing the expression differences that PT- and MT-specific genes showed between the two surrounding benign epithelial cells, we noted that the majority of PT-specific genes were higher expressed in healthy colon compared to healthy liver, while MT-specific genes were more highly expressed in healthy liver compared to healthy colon (Figure 3A). Thus, the majority of differences in gene expression between the primary and secondary tumors are reflected in the surrounding tissue, strengthening the hypothesis that the surrounding tissues may drive these expression differences in addition to those due to the metastatic process itself.

To systematically identify tissue-adaptive candidate genes, we implemented a novel approach utilizing a two-way comparison to identify expression differences between the MT and the PT cells that also reflect differences in the expression profile of normal liver versus normal CE cells (Figures 3B and S1, see STAR Methods). We termed a gene “adaptive” either if its expression was higher in the MT relative to the PT *and* higher in the LE compared to the CE cells, or alternatively, if the gene was lower expressed in the MT *and* accordingly lower expressed in healthy liver compared to healthy colon. We computed respective expression fold changes per donor (to control for donor-specific expression patterns), and subsequently classified genes to be PT and CE (PTCE) specific or MT and LE (MTLE) specific (Figure 3B and Table S6). We required that candidate genes we termed “specific” showed consistent expression differences across multiple tissue samples from different donors (see STAR Methods). Some genes were tissue-exclusive, i.e., undetectable in some of the tissues, but expressed at relevant levels in others (Table S4, see STAR Methods). This analysis was limited to donors with the cell types of interest available in our data (see STAR Methods for details).

Out of the 5,019 (98.5% protein coding) genes analyzed, e.g., detected in all four cell types (Figure S3C, see STAR Methods), 550 (i.e., ∼11%) had consistent expression changes across donors (Figures 3C, S3D, and S3E). Of those, the vast majority were either consistently upregulated in the PTCE cells (292 PTCE genes), or in the MTLE cells (172 MTLE genes, Figures 3C and 3D). Thus, these 464 genes (84% of 550 with a consistent expression pattern) had expression changes in the MT qualitatively matching expression differences in the respective healthy host tissues. Only a small fraction of genes consistently (i.e., across donors) changed their expression in opposing directions (49 genes upregulated in PT and LE, 37 genes upregulated in MT and CE). Overall, the distribution of the candidate genes differed significantly from (a) a random distribution (*p* value <0.001, Pearson’s chi-squared test) and (b) the non-candidate genes (*p* value <0.001, Pearson’s Chi-squared Test). This result revealed an alignment between the expression patterns of tumor cells and their surrounding tissue. We note that the PTCE and MTLE genes did not contain all PT and MT-specific genes defined above (Figure 2B) due to small differences in which genes are included in the analysis (see STAR Methods). These changes in the preprocessing, however, only result in minimal differences in the analysis and do not change the overall fold changes (Figures S4A–S4C). The expression fold changes in the tumor cells (MT versus PT) were on average smaller than the fold changes in the healthy tissues (LE versus CE; Figure 3C). This finding may suggest that metastatic cells move toward a “liver-like” state, but do not fully adopt the expression program of LE cells.

### Validation of tissue adaptive candidate genes

To validate PTCE and MTLE candidate genes in external cohorts, we utilized an (1) external scRNA-seq dataset with primary colon tumor and matching liver MT cells^34^ containing 23,954 cells from five donors and (2) a bulk gene expression dataset with primary colon tumor and liver metastasis samples from 334 donors.

**Figure 4:**
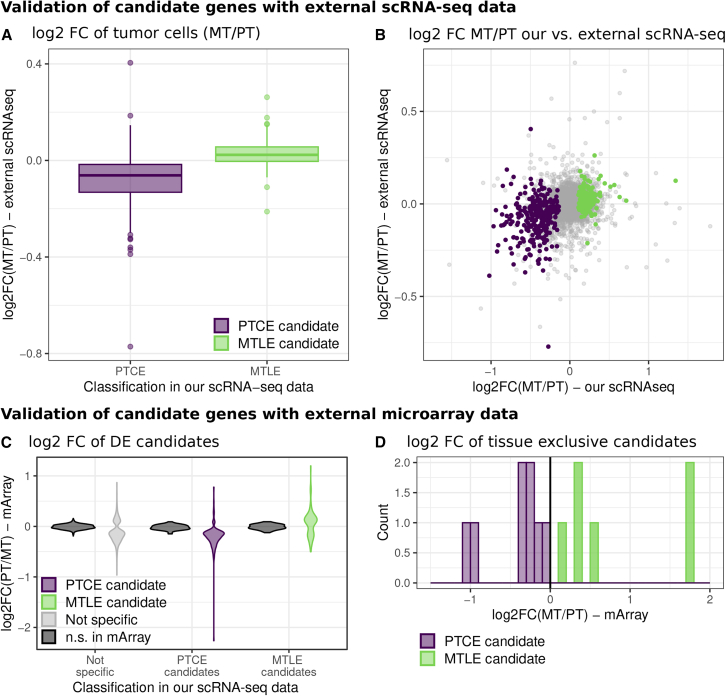
Validation of MTLE and PTCE candidate genes with external data (A) Log2-fold changes (FC) from metastasis to primary tumor cells in external scRNA-seq data (23,954 cells from 5 donors) plotted by their classification in our data (PTCE candidates = purple, MTLE candidates = green). Box limits represent the interquartile range (IQR; 25th to 75th percentiles), the solid line inside the box indicates the median, and the whiskers extend to data points within (1.5 × IQR) from the quartiles. Outliers are not shown. (B) Log2 FC from liver to colon epithelial cells in our scRNA-seq and the external scRNA-seq data plotted against each other. Colors as in a) with non-specific genes being depicted in gray. (C) Log2 FC from metastasis to primary tumor samples of external microarray data (334 donors) stratified by the gene classification in Figure 3C. Fold changes of genes that were not differentially expressed in the microarray dataset (adjusted *p* value <0.05) are shown in black; other colors as in a). (D) Log2 FC from metastasis to primary tumor samples of external microarray data of all genes that have been found to have tissue-exclusive expression in our data. Colors as in A).

We analyzed the external scRNA-seq dataset by applying the same analysis pipeline as above and retrieved pseudo-bulk log fold changes between the metastatic and PT cells (see STAR Methods; Figure S5A). All genes detected in both the internal and external scRNA-seq data showed highly consistent expression patterns in the external scRNA-seq data, when compared with our earlier findings. 105 out of 151 MTLE genes (70%) were more highly expressed in the MT compared to the PT, and 223 out of 271 PTCE genes (82%) were more highly expressed in the PT compared to the MT (Figures 4A and 4B).

The bulk gene expression dataset obtained from 334 donors^63^^,^^64^^,^^65^^,^^66^^,^^67^ (184 primary colon carcinomas, 150 liver metastases; Figures S5B and S5C) further validated our MTLE and PTCE genes. Genes with a differential expression in our scRNA-seq and a significant differential expression in the bulk data showed highly consistent directions of effects for both the PT and MT candidate genes (Figure S5D; 607 out of 909 genes that were detected in our scRNA-seq data and in the bulk measurements had effects in the same direction), as well as the PTCE and MTLE genes (Figure 4C; 321 out of 430 genes measured in both datasets had effects in the same direction; Tables S4 and S6). Note that tissue-exclusive genes were also confirmed by this analysis (Figures 4D and S5E). Hence, our analysis identified hundreds of genes whose expression in metastases adapts to the liver environment consistently across three cohorts.

To further validate tissue-adaptive gene candidates, we performed *in vitro* co-cultivation experiments (see STAR Methods for details). Two human CRC cell lines (HCT116 and HT29) were co-cultured with four hepatocyte-like cell lines (Huh7, HepG2, Hep3B, PH5CH8) and one non-tumorigenic colonic cell line (NCM460). Subsequently, twelve MT or MT-and-liver-epithelial candidate genes, chosen based on signal strength and consistency within our cohort and external datasets, were quantified in the co-cultured CRC cells by real-time PCR (qPCR). The selected candidates included genes with plausible links to liver-associated biology and epithelial state changes, including *FABP1* and *MTTP*, which are associated with hepatic fatty acid handling and apoB-containing lipoprotein assembly,^68^^,^^69^ respectively, *TM4SF4*, encoding a tetraspan membrane glycoprotein described in intestinal epithelium and liver,^70^ and *DCDC2*, which emerged consistently in our datasets and has been linked to microtubule- and primary cilia-associated biology, with additional reports suggesting a role in hepatobiliary biology.^71^^,^^72^ To our knowledge, *DCDC2* has not been described as a CRC-liver MT adaptation gene before.

Among the analyzed candidates, *FABP1* and *DCDC2* showed the clearest support in HCT116 cells under hepatocyte-like co-culture conditions relative to NCM460. In HT29 cells, the overall response was weaker, with *DCDC2* showing the most consistent support and *FABP1* showing partial support. The remaining candidates did not show consistent statistically supported induction in the individual condition-wise comparisons. Corresponding *p* values for all analyzed genes are provided in extended data Figure S6. Together, these results provide additional support that at least part of the observed expression changes during metastatic adaptation is influenced by tissue context.

### Cancer cells adapt transcriptional networks to their environment

To move beyond single genes and identify entire transcriptional networks affected by the tissue adaptation, we performed an analysis of pre-annotated molecular pathways (BioCarta,^73^^,^^74^ KEGG,^75^^,^^76^ PID^77^ and Reactome,^78^^,^^79^ see STAR Methods). Using fast gene set enrichment analysis^80^ (FGSEA) we determined qualitative effect directions of all pathways for each pairwise cell type and donor comparison; that is, we determined if the pathway was more highly expressed in MT versus PT *and* if it was more highly expressed in LE versus CE in each donor. Subsequently, we defined pathways as “consistent” if the effect directions of the normalized enrichment scores (NESs) of both the MT-versus-PT and LE-versus-CE comparison were the same across all donors included in the analysis. Hence, expression differences did not have to be statistically significant within individual donors, as long as effect directions were consistent across donors. Of all pathways included in our analysis (*n* = 1103), 436 were “consistent” according to this definition (Table S7). Those 436 pathways showed a strong signal toward being upregulated either concordantly in the PT and colon epithelium (*n* = 234), or in the MT and liver epithelium (*n* = 147). Only very few pathways were consistently altered in the cross-tissue pairs (higher in PT and LE cells: 33, higher in MT and CE cells: 22). The *p* value for the quadrant distribution of pathways with the NES agreeing in directionality between donors and cell type comparisons is <0.001 (Fisher’s exact test). These findings are in line with our previous analyses (Figures 3A and 3C) and confirm that differences in expression patterns observed between the PT and MT are, to a large extent, also observed in the epithelial cells. Hence, these changes are likely due to cellular pathways that are differentially regulated depending on the tissue context.

**Figure 5:**
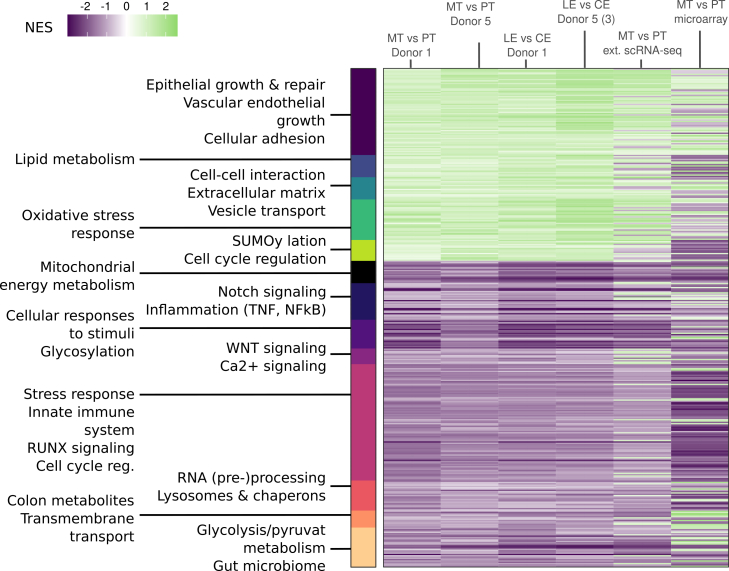
Overview of pathways shows adaptive expression signature in PT or MT and their respective surrounding tissue Normalized enrichment scores (NESs), i.e., the enrichment score normalized to the mean enrichment of random samples of the same size. Pathways shown here have consistent directionality between MT & PT and LE & CE in our scRNA-seq data. Pathways were clustered based on similarity, i.e., grouping together pathways containing similar genes.

By merging largely overlapping pathway annotations, we identified five groups of pathways being higher expressed in metastases and liver epithelium and eight groups of pathways being higher expression in PTs and CE cells (Figure 5). Biological processes involved in epithelial growth and cellular adhesion as well as extracellular matrix, cell-cell interaction, and vesicular transport are more highly expressed in the metastases and epithelial liver tissue. Among the pathways in the area of ECM and cell-cell adhesion are well-known liver pathways like the *Arf6 pathway*,^81^ that couples HFG signaling to hepatocyte proliferation and liver regeneration^82^ and *heparan sulfate/heparin metabolism*^83^^,^^84^^,^^85^^,^^86^ while they also include more generic functions, e.g., RhoA activity^87^ or RhoB GTPase cycle.^88^ The adaptation of these pathways may be a requirement for the successful establishment of the MT in the liver environment. Other pathways displaying a higher expression in MT and liver include different metabolic processes such as lipid metabolism and oxidative stress response.

**Figure 6:**
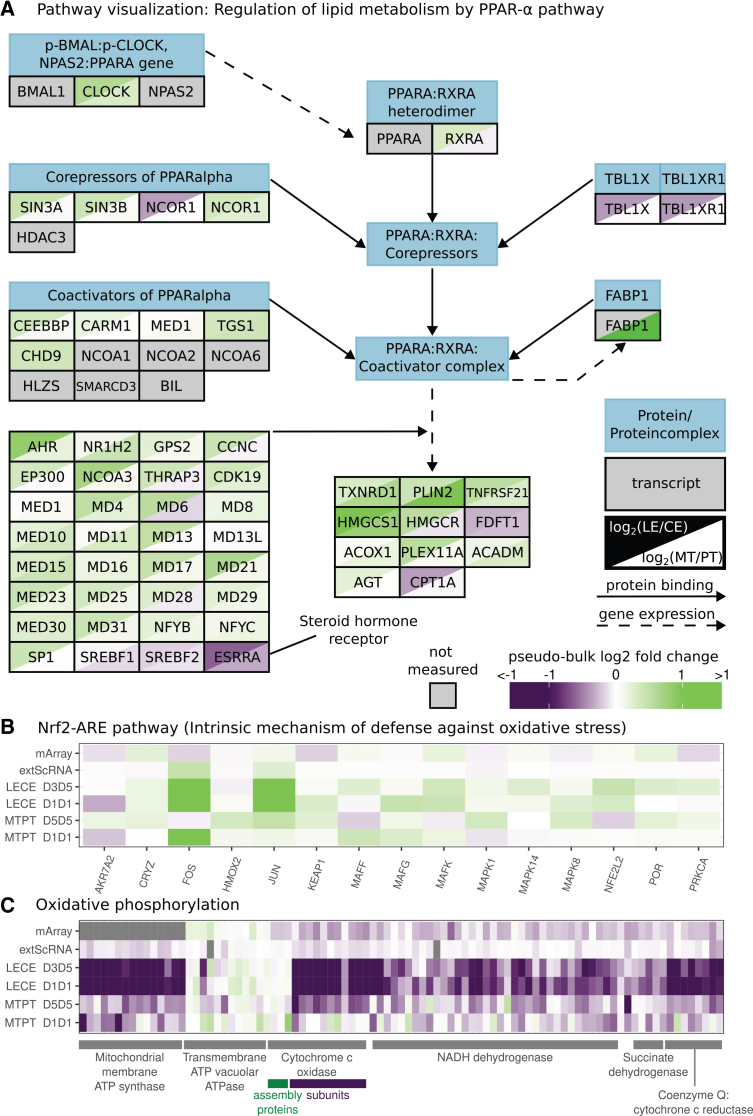
Molecular pathways adapt to the host tissue in cancer cells (A) Selected members of the pathway “Regulation of lipid metabolism by PPAR-α” (Reactome), which is highly expressed in the liver metastasis and liver epithelial cells of our scRNA-seq data. Average log2-fold changes between the metastasis and primary tumor cells (MT/PT) as well as the liver and colon epithelial cells (LE/CE) are displayed. Color: gene expression log2-fold change of the liver versus colon epithelial cells (LE/CE; upper part) as well as for the metastasis versus the primary tumor cells (MT/PT; lower part). Arrows indicate protein binding or gene expression. All transcripts of the pathway detected in our scRNA-seq data are visualized. (B) Average log2-fold changes for the “Nrf2-ARE pathway” (Biocarta), which is higher expressed in the metastasis and liver epithelial cells. Comparisons are indicated on the left (rows) and genes at the bottom (columns). Color code as in a). (C) As in (B) but for the KEGG pathway “Oxidative phosphorylation” (KEGG), which is higher expressed in the primary tumor and colon epithelial cells.

Particularly noteworthy in this context is the pathway *Regulation of lipid metabolism by PPAR-α*^89^ that was consistently higher expressed in the MT and LE cells compared to the PT and colon epithelium (Figure 6A). Importantly, these expression differences were also validated in the external bulk- and single-cell expression data (Figure S7A). PPAR-α is a major regulator of lipid metabolism in the liver, specifically regulating fatty acid oxidation.^50^^,^^51^ Further, the activation and expression of PPAR-α have been associated with tumorigenesis in CRC, as well as worse outcomes in liver MT of CRC.^90^^,^^91^ Pathway activity analysis revealed an increase in activity from CE to PT cells and a further increase in MT with the highest activity present in the LE cells (Figure S7B). Thus, PPAR-α may be an important contributor to the adaptation of the tumor to the liver-specific lipid metabolism and establishing itself as a MT. Another pathway consistently higher expressed in the MTLE in both the scRNA-seq and microarray data is the *Nrf2-ARE* pathway, an intrinsic mechanism of defense against oxidative stress^92^ (Figures 6B and S7C). The nuclear factor erythroid 2-related factor 2 (Nrf2) has been identified as both a tumor suppressor and an oncogene, as it can protect benign cells from oxidative stress^93^ as well as help cancer cells cope with high levels of reactive oxygen species (ROS).^93^

Two of the pathway groups higher expressed in the PT and colon epithelium are closely linked to the function of the colon: colon metabolite and transmembrane transport-related pathways and pathways connected to glycolysis/pyruvate metabolism and gut microbiome (Figure 5). For example, we find that *propanoate metabolism*^59^ is higher expressed in PT and colon epithelium, a pathway that reflects colon-specific host metabolic responses to microbiome-derived metabolites. Our previous finding, that genes related to *mitochondrial energy metabolism*^94^ are upregulated in the PT compared to the MT (Figures 2C and 2D) can also be observed in the CE compared to the LE cells, pinpointing to a context-driven energy metabolism of cancer cells. We could validate these results in independent scRNA-seq and microarray data (Figures 6C and S7D). These findings suggest that the shift in energy metabolism from oxidative phosphorylation to glycolysis commonly observed in liver MT may result from a metabolic adaptation to the new environment. Interestingly, while all detected subunits of the mitochondrial membrane ATP synthase (F1F0 ATP Synthase) were higher expressed in the cells derived from the colon site, the opposite was true for the vast majority of subunits of the transmembrane ATP vacuolar ATPase (V-ATPase). The F1F0 ATP Synthase generates ATP using a proton gradient, and hence an increase in its subunits reflects an increase in energy availability through the mitochondria.^95^ This may be a cellular adaptation to environmental changes such as alterations in nutrient availability, oxygen levels, or other external stress factors.^96^^,^^97^ V-ATPases, on the other hand, acidify intracellular compartments by transporting proteins across the plasma membrane using ATP and play a fundamental role in maintaining pH homeostasis.^98^^,^^99^ Therefore, they are involved in various physiological and pathological processes, such as macropinocytosis, autophagy, cell invasion, and cell death. In this context, overexpression of a V-ATPase subunit and its regulators have been identified to being closely related to tumor invasion and MT, offering an explanation why we find the subunits of the V-ATPase being higher expressed in the LE and MT cells.^99^^,^^100^

## Discussion

The formation of metastases is the main cause of cancer-related death. The manifestation of metastases of a solid tumor requires cancer cells to change their characteristics and behavior, migrate out of the PT, intra- and extravasate blood vessels, and eventually proliferate in a different tissue.^1^^,^^2^^,^^3^ Importantly, such adaptation may not require specific mutations to rewire metabolic networks. Instead, external cues, such as the availability of nutrients, oxygen, and signaling molecules, may suffice to adapt their metabolism.^27^^,^^101^^,^^102^

We present a novel concept for the identification of tissue-adaptive gene expression changes that is generally applicable to metastases established from solid tumors. Here, the quadruple-paired cohort at the single-cell level is essential to retrieve the full picture of metastatic adaptations and organotropism. Single-cell data enable us to retrieve tissue-adaptive expression patterns based on specific cell types; therefore, tumor signals are free of tumor purity concerns. The application of this concept to untreated, paired samples from donors with CRC and liver MT revealed tissue-adaptive expression changes in the tumor cells. We validated our findings on published single-cell and bulk expression datasets, showing that the detected adaptive expression pattern reproducibly occurs in liver metastases originating from primary colon cancer.

We have deliberately focused our analyses on the transcriptional adaptations in the cancer cells, identified tissue-adaptive expression changes in two-dimensional transcriptomic space based only on epithelial cells, and restricted our *in vitro* validation to epithelial cell types. We did not consider the TME, as this has been investigated earlier.^35^ The TME, including immune cells and different types of fibroblasts, is important for tumor biology and the formation of metastases and needs to be considered for a holistic view of metastatic spread. It has been shown in mice, for example, that a progressive increase of myeloid and exhausted T cells and a decrease of T/NK and B cells form a pre-metastatic niche before visible metastases establish and that an early increase of a subpopulation of neutrophils is associated with poor prognosis.^103^ While the emphasis on the epithelial rather than the TME compartment is a limitation of our study, it allows us to focus on the cancer-cell specific aspect of adaptation.

The upregulation of liver-specific pathways, i.e., pathways that are more highly expressed in the LE compared to CE cells such as the PPAR-*α* pathway or the insulin signaling pathway, in liver metastases indicates metabolic adaptation. We also find colon-specific pathways e.g., pathways that reflect host metabolic responses to microbiome-derived metabolites, being highly expressed in the primary colon tumor but not in the liver MT. Taken together, this indicates an adaptation of the expression pattern driven by external cues, which is consistent with the notion that the establishment of distant metastases from a primary colon tumor may not require *de novo* mutations.^102^^,^^104^ Commonly reported differences in the expression pattern between the PT and MT of specific cancer types, such as the switch from oxidative phosphorylation toward glycolysis in liver MT compared to primary colon tumor,^27^^,^^46^^,^^47^ may be driven by tissue adaptive processes in the MT. The fact that only a small fraction of circulating tumor cells actually establishes a MT, makes it likely that the metabolic adaptation results from an interplay of genetic and epigenetic predisposition with signaling responses to external cues.^101^

Our results strongly suggest that an adaptation of metastatic expression patterns supports the successful integration into the new host environment. Our findings are in line with a previous study where the up-regulation of liver-specific genes has been reported for CRC based on bulk transcriptomics with liver-specific transcription factors FOXA2 and HNF1A binding to altered chromatin structures in liver metastases.^32^ Epigenetic changes with accessible chromatin specifically gained in liver metastases showed enrichment for HNF4A binding sites in a xenograft model with patient-derived cell lines.^33^ We confirm the overall process of adaptation toward a liver-like phenotype using single-cell transcriptomics of primary cells of patients with CRC. Metabolic changes are a dominant feature of this adaptation, likely contributing to the pathogenicity of a tumor. For example, the upregulation of the *Nrf2-ARE* pathway that we have observed in this study may result from generally higher ROS levels in the liver compared to the colon, which is known to further increase the pathogenicity and treatment resistance of the MT.^105^^,^^106^^,^^107^ Similarly, the activation and expression of PPAR-α, which is also classified as a tissue-adaptive process in our study, is associated with tumorigenesis in CRC, as well as worse outcomes in liver MT of CRC,^90^^,^^91^ resulting in PPAR-α being a repeatedly discussed cancer drug candidate.^108^^,^^109^ Hence, targeting tissue-adaptive processes may not only prevent metastasization, but also address treatment resistance.

Our co-culture experiments provide an *in vitro* validation of the context-driven adaptation of tumor expression patterns by recapitulating the effect of hepatic versus colonic cellular context on CRC gene expression. In this system, hepatocyte-like co-cultures were sufficient to reproduce parts of the liver-associated expression changes observed in donor-derived samples, supporting a contribution of tissue context to transcriptional adaptation. As the experiment does not capture the full complexity of the metastatic microenvironment, including stromal, immune, and extracellular matrix-derived influences, these results provide supportive evidence for context-dependent transcriptional adaptation but do not offer a comprehensive functional model of metastatic colonization.

We present a novel approach that enables us to deduce tissue-adaptive expression changes in MT, providing a rare and useful dataset of treatment-naive quadruple-paired scRNA-seq from donors with CRC and liver MT. These adaptations potentially increase the pathogenicity of the metastatic cells and may provide new therapeutic strategies based on inhibiting the metabolic adaptation of cancer cells to their host environment. The general applicability of the approach presented here provides an opportunity for generating much deeper insight into the etiology of MT formation and the mechanisms determining treatment response way beyond CRC.

### Limitations of the study

Our findings should be interpreted in light of several limitations. Most profoundly, our study is limited by the small number of scRNA-sequenced donors available, as we are relying on quadruple samples from treatment naive patients. The number of donors available in this cohort is not large enough to estimate *p* values based on pseudo-bulk expression patterns; therefore, our analysis largely relies on consistent directionality of log2-fold changes between tissues and donors. The strict requirement, that the directionality of every gene has to be conserved across all tissues and donors, may not be transferable to potentially larger cohort studies. Ideally, a larger cohort would enable the estimation of *p* values as a selection criterion for tissue-adaptive genes or a different threshold for the percentage of donors having to agree if the signal may be set (e.g., 80% instead of the current 100%). Due to these limitations, the observations we make in our cohort are descriptive. As another limitation to this work, we are studying metastatic adaptation exclusively on the level of gene expression and therefore capture transcriptional adaptation rather than the full functional state of the cell. Changes at the mRNA level do not necessarily translate directly into corresponding protein abundance or activity, as post-transcriptional regulation, protein turnover, and context-dependent signaling may substantially modify the cellular phenotype. Our results should therefore be interpreted as evidence for transcriptional reprogramming associated with metastatic adaptation, while the corresponding proteomic and functional consequences remain to be addressed in future work.

## Resource availability

### Lead contact

Requests for further information and resources should be directed to and will be fulfilled by the lead contact, Axel M. Hillmer (ahillmer@uni-koeln.de).

### Materials availability

This study did not generate new unique reagents.

### Data and code availability

• The single-cell RNA-seq data have been deposited at the European Genome Phenome Archive (EGA) under accession no. EGAS50000000817. Corresponding preprocessed count matrices are available under https://zenodo.org/records/14794206.
• Code for all analysis performed and the corresponding figures is provided on GitHub: https://github.com/beyergroup/Gene-expression-adaptation-of-metastases.
• Any additional information required to reanalyze the data reported in this paper is available from the lead contact upon request.

**Table:** STROBE Statement—Checklist of items that should be included in reports of cohort studies ∗Give information separately for exposed and unexposed groups

	Item No	Recommendation	Page No
**Title and abstract**	1	(*a*) Indicate the study’s design with a commonly used term in the title or the abstract	2
		(*b*) Provide in the abstract an informative and balanced summary of what was done and what was found	2
**Introduction**			
Background/rationale	2	Explain the scientific background and rationale for the investigation being reported	3–5
Objectives	3	State specific objectives, including any prespecified hypotheses	5
**Methods**			
Study design	4	Present key elements of study design early in the paper	5,6,20
Setting	5	Describe the setting, locations, and relevant dates, including periods of recruitment, exposure, follow-up, and data collection	20
Participants	6	(*a*) Give the eligibility criteria, and the sources and methods of selection of participants. Describe methods of follow-up	20
		(*b*) For matched studies, give matching criteria and number of exposed and unexposed	N/A
Variables	7	Clearly define all outcomes, exposures, predictors, potential confounders, and effect modifiers. Give diagnostic criteria, if applicable	Table S1
Data sources/measurement	8∗	For each variable of interest, give sources of data and details of methods of assessment (measurement). Describe comparability of assessment methods if there is more than one group	20–24
Bias	9	Describe any efforts to address potential sources of bias	22–23
Study size	10	Explain how the study size was arrived at	15,20
Quantitative variables	11	Explain how quantitative variables were handled in the analyses. If applicable, describe which groupings were chosen and why	20–24
Statistical methods	12	(*a*) Describe all statistical methods, including those used to control for confounding	20–24
		(*b*) Describe any methods used to examine subgroups and interactions	22–24
		(*c*) Explain how missing data were addressed	18,22-24
		(*d*) If applicable, explain how loss to follow-up was addressed	N/A
		(*e*) Describe any sensitivity analyses	N/A
**Results**			
Participants	13∗	(a) Report numbers of individuals at each stage of study—eg numbers potentially eligible, examined for eligibility, confirmed eligible, included in the study, completing follow-up, and analyzed	6,9,10, 22-24
		(b) Give reasons for non-participation at each stage	18, 22-23
		(c) Consider use of a flow diagram	N/A
Descriptive data	14∗	(a) Give characteristics of study participants (e.g., demographic, clinical, social) and information on exposures and potential confounders	Table S1
		(b) Indicate number of participants with missing data for each variable of interest	N/A
		(c) Summarise follow-up time (e.g., average and total amount)	N/A
Outcome data	15∗	Report numbers of outcome events or summary measures over time	6,9,10, 22-24
Main results	16	(*a*) Give unadjusted estimates and, if applicable, confounder-adjusted estimates and their precision (e.g., 95% confidence interval). Make clear which confounders were adjusted for and why they were included	N/A
		(*b*) Report category boundaries when continuous variables were categorized	22–24
		(*c*) If relevant, consider translating estimates of relative risk into absolute risk for a meaningful time period	N/A
Other analyses	17	Report other analyses done—eg analyses of subgroups and interactions, and sensitivity analyses	N/A
**Discussion**			
Key results	18	Summarise key results with reference to study objectives	13–15
Limitations	19	Discuss limitations of the study, taking into account sources of potential bias or imprecision. Discuss both direction and magnitude of any potential bias	15
Interpretation	20	Give a cautious overall interpretation of results considering objectives, limitations, multiplicity of analyses, results from similar studies, and other relevant evidence	15
Generalisability	21	Discuss the generalisability (external validity) of the study results	14,15
**Other information**			
Funding	22	Give the source of funding and the role of the funders for the present study and, if applicable, for the original study on which the present article is based	16

Note: An Explanation and Elaboration article discusses each checklist item and gives methodological background and published examples of transparent reporting. The STROBE checklist is best used in conjunction with this article (freely available on the Web sites of PLoS Medicine at http://www.plosmedicine.org/, Annals of Internal Medicine at http://www.annals.org/, and Epidemiology at http://www.epidem.com/). Information on the STROBE Initiative is available at http://www.strobe-statement.org.

## Acknowledgments

The study was funded by the 10.13039/501100001659Deutsche Forschungsgemeinschaft (10.13039/501100001659DFG, 10.13039/501100001659German Research Foundation) with INST 216/1063-1 FUGG (project no. 446411360) and Collaborative Research Center CRC1310 “Predictability in Evolution” (subprojects C2 and C4). We thank David Pertzborn, Anna Mühlig, and Daniela Pelzel of the University Hospital Jena for discussion and support on validation work.

## Author contributions

Conceptualization: L.N., A.B., and A.M.H.; data curation: L.N., M.W., and M.K.; formal analysis: L.N., M.K., and M.W.; funding acquisition: A.B., A.M.H.; investigation: S.H., M.K., L.N., M.F., A.Q., A.H., I.J., and S.S., methodology: L.N., S.H., and M.K.; project administration: A.M.H., A.B.; resources: P.S.P., R.W., M.B., C.J.B., A.B., and A.M.H.; software: L.N., M.W.; supervision: A.B., A.M.H.; validation: L.N., S.H., and M.K.; visualization: L.N.; writing – original draft: L.N., S.H., A.B., and A.M.H.; writing – reviewing and editing: L.N., M.K., A.M.H., and A.B.

## Declaration of interests

The authors declare no competing interests.

## Declaration of generative AI and AI-assisted technologies in the writing process

No artificial intelligence tools were used in the study and for the preparation of the manuscript.

## STAR★Methods

### Key resources table

**Table:** 

REAGENT or RESOURCE	SOURCE	IDENTIFIER
**Antibodies**		

PE/Cy7-conjugated anti-human CD45 antibody	BioLegend	Cat# 304015; clone HI30; RRID:AB_314403
Anti-human CD326 (EpCAM) FITC, clone HEA-125	Miltenyi Biotec	Cat# 130-113-263; RRID:AB_2726064

**Bacterial and virus strains**		

EF1a-Luciferase (firefly)-2A-RFP (Neo) concentrated lentivirus	GenTarget	Cat# LVP441-PBS

**Biological samples**		

Primary colon tumor (PT), normal colon (NC), liver metastasis (MT), and normal liver (NL) from donors with colorectal cancer and liver metastasis	University Hospital Cologne	Institutional review board of the University of Cologne: 21-1467 and 21-1382_2

**Chemicals, peptides, and recombinant proteins**		

DNase I (500 U·mL^-1^)	AppliChem PanReac	Cat# A3778
Collagenase IV (320 U·mL^-1^)	Thermo Fisher Scientific	Cat# 17104019
Dispase II (2 U·mL^-1^)	Sigma-Aldrich	Cat# D4693-1G
Fetal bovine serum (FBS)	Capricorn Scientific	Cat# AN-22643
RPMI-1640 medium	Life Technologies GmbH	Cat# 61870044
Dimethyl sulfoxide (DMSO)	PAN-Biotech	Cat# P60-36720100
Propidium iodide	Thermo Fisher Scientific	Cat# P1304MP
Penicillin-streptomycin	Life Technologies GmbH	Cat# 15140122
DMEM	Life Technologies GmbH	Cat# 41965062

**Critical commercial assays**		

Chromium Single Cell 3ʹ Solution	10x Genomics	Chromium Next GEM Single Cell 3ʹ Kit v3.1, 16 rxns; Cat# PN-1000268
Maxwell® RSC simplyRNA Tissue Kit	Promega	Cat# AS1340
GoScript™ Reverse Transcription Mix, Random Primers	Promega	Cat# A2800
GoTaq® qPCR Master Mix	Promega	Cat# A6001
NovaSeq 6000 S2 Reagent Kit v1.5 (100 cycles)	Illumina	Cat# 20028316

**Deposited data**		

scRNA-seq data generated in this study	European Genome-Phenome Archive (EGA)	EGAS50000000817
Preprocessed count matrices generated in this study	Zenodo	https://zenodo.org/records/14794206
External scRNA-seq dataset	GEO	GSM7058755
External microarray datasets	GEO	GSE79959; GSE96528

**Experimental models: Cell lines**		

HCT116_Luc_RFP	Gift from external working group; transduced in this study	HCT 116 derivative expressing luciferase and RFP; parent RRID:CVCL_0291
HT29_Luc_RFP	ATCC; transduced in this study	HT-29 (ATCC HTB-38) derivative expressing luciferase and RFP; parent RRID:CVCL_0320
Huh-7	Gift from external working group	RRID:CVCL_0336
HepG2	Gift from external working group	RRID:CVCL_0027
Hep3B	Gift from external working group	Hep 3B2.1-7; RRID:CVCL_0326
PH5CH8	Gift from external working group	RRID:CVCL_VL00
NCM460	Cytion	RRID:CVCL_0460

**Oligonucleotides**		

Validated primer sets for candidate genes and HPRT1	This study	See Table S9

**Software and algorithms**		

Cell Ranger v6.1.2	10x Genomics	RRID:SCR_017344
Seurat v4.0.3	Satija Lab	RRID:SCR_016341
R	R Foundation for Statistical Computing	RRID:SCR_001905
R package fgsea (v1.31.0)	Bioconductor	RRID:SCR_020938
R package inferCNV	Broad Institute	RRID:SCR_021140
R package limma (v3.58.1)	Bioconductor	RRID:SCR_010943
R package oligo (v1.68.1)	Bioconductor	N/A
R package GSVA (v2.2.1)	Bioconductor	RRID:SCR_021058
R package MASS (v7.3-61)	CRAN	N/A
MAST	Bioconductor	RRID:SCR_016340
PanglaoDB	Database	RRID:SCR_022580
MSigDB canonical pathways (BioCarta, KEGG, PID, Reactome)	UC San Diego / Broad Institute	RRID:SCR_016863
GitHub repository for analysis code	GitHub	beyergroup/Gene-expression-adaptation-of-metastases
Code for all analysis performed and the corresponding figures	This paper	https://github.com/beyergroup/Gene-expression-adaptation-of-metastases

**Other**		

GentleMACS Dissociator	Miltenyi Biotec	Cat# 130-093-235; RRID:SCR_020267
BD FACSAria Fusion cell sorter	BD Biosciences	RRID:SCR_025715
Sony MA900 cell sorter	Sony Biotechnology	RRID:SCR_026300
Chromium X platform	10x Genomics	RRID:SCR_024537; Accessory Kit Cat# PN-1000331
NovaSeq 6000 sequencer	Illumina	Cat# 20012850; RRID:SCR_016387
NanoDrop 2000 Spectrophotometer	Thermo Fisher Scientific	RRID:SCR_018042
CFX96 Touch Real-Time PCR Detection System	Bio-Rad	RRID:SCR_018064
T75 tissue culture flask	Greiner Bio-One	Cat# 658175
100-μm cell strainer	Corning	Cat# 431752

### Experimental model and study participant details

#### Human subject statement

The study was approved by the institutional review board of the University of Cologne (21-1467 and 21-1382_2). Informed consent was obtained from all subjects. Experiments conformed to the principles set out in the Declaration of Helsinki and the Department of Health and Human Services Belmont Report.

#### Sample collection and processing

Primary colon tumor (PT), normal colon (NC), liver metastasis (MT) and normal liver (NL) samples were collected from five donors with colorectal primary tumors and liver metastasis in the years 2019 and 2020 at the University Hospital of Cologne (Table S1). Donors were required to have colorectal cancer with liver metastases and to be treatment naïve. The inclusion criteria limited the study to five donors which was considered sufficient to investigate the fundamental biological question. All primary tumors were located at the colon sigmoideum or near rectum, except for donor 1 (D1), which had two primary tumors, one located in the sigmoideum and one in the ascending colon. The following samples were collected from each donor: D1: two PT, NC, MT, NL; D2: PT, NC; D3: MT, NL; D4: PT, NC, MT, NL; D5: PT, NC, MT, NL. Preparations of colon ascendence (PT) of D1 and MT of D4 resulted in too few cells for reliable analysis and were excluded after quality control from the study. The samples were processed in three batches (Batch 1: D1, Batch 2: D2 & D3, Batch 3: D4 & D5) – all samples within a batch were processed, FACS sorted and sequenced together. Due to sample size of three male and two female patients, no attempts were made to identify sex or gender-specific effects.

Fresh tissue samples from surgical resectates or endoscopies were dissociated as described earlier.^110^ In brief, the tissue was minced to small pieces, disrupted in a C-tube used with the GentleMACS Dissociator (Miletnyi Biotec) combined with enzymatic digestion with DNAse I (500 U·mL−1; AppliChem PanReac), collagenase IV (320 U·mL−1; Thermo Fisher Scientific), and dispase II (2 U·mL−1; Sigma-Aldrich), filtered through a 100-μm cell strainer, collected and resuspended in 60% RPMI-1640 medium (Thermo Fisher Scientific), 30% FBS (Capricorn Scientific), and 10% dimethyl sulfoxide (DMSO) (Sigma-Aldrich) for freezing at -80°C. After 24 hours samples were transferred to liquid nitrogen until fluorescence activated cell sorting (FACS).

### Method details

#### FACS and single-cell sequencing

Single-cell suspensions were stained with PE/Cy7-conjugated antibodies against CD45 (Biolegend) as a leukocyte marker to reduce leukocytes and with propidium iodide (Thermo Fisher Scientific) to distinguish live and dead cells according to the manufacturer's specifications. Flow cytometry assisted cell sorting was performed on BD FACSAria Fusion (BD Biosciences) using a 100-μm nozzle and a LE-MA900FB (Sony) with a 100- μm chip. For each sample, 10.000 living cells were sorted, comprising 30% of CD45-positive cells and 70% of CD45-negative cells, whenever applicable. For samples of D4 and D5, an additional staining for EpCAM using CD326 (EpCAM) FITC Antibody anti-human, clone HEA-125 (Miltenyi Biotec), was performed to further enrich for epithelial cells aiming at a ratio of ⅓ CD45^+^, ⅓ EpCAM+, and ⅓ CD45-EpCAM- cells. Collected single cells were placed on ice and further processed using the Chromium™ Single Cell 3’ Solution (10x Genomics) and sequenced on a NovaSeq 6000 (Illumina) with Illumina 3’ v3.1-paired end chemistry, 29-10-10-89 bp aiming at 25,000 read pairs per cell. Targeted cell recovery was aiming at 3000 cells per sample.

#### Co-culture of cancer cells with hepatic and colonic epithelial environment

Co-cultures between luciferase- and RFP-labeled CRC cell lines (HCT116_Luc and HT29_Luc) and either hepatic cell lines (Huh7, HepG2, Hep3B, PH5CH8) or the non-transformed colonic epithelial cell line NCM460 were established (1:1 ratio). Cell lines were regularly tested for contamination by mycoplasma using an in-house PCR assay. NCM460 and HT29 were purchased for this study and have not been further validated. HCT116, Huh-7, HepG2, Hep3B, and PH5CH8 have been monitored for morphology over time but not re-genotyped for the present study. Co-cultures were performed in T75 tissue culture flasks (75 cm^2^ surface area), with a seeding density of 2 × 10^6^ cells per cell line. For HepG2 and NCM460, 1.3 × 10^6^ cells were used per cell line due to limited availability. Cells were incubated for 72 h at 37°C and 5% CO_2_ in complete DMEM supplemented with 10% fetal bovine serum (FBS) and 1% penicillin-streptomycin. A single standardized culture medium was used across all co-culture conditions to allow direct comparison between hepatic and colonic reference settings during the assay period. This experimental design was chosen to compare directional effects of hepatic versus colonic epithelial context on CRC cell gene expression under controlled *in vitro* conditions.

After co-culture, cell monolayers were visually inspected to confirm sufficient confluency (>70% for most conditions). HepG2 co-cultures showed lower confluency, consistent with their compact growth pattern and reduced proliferation rate (data not shown). CRC cells were isolated from mixed populations via fluorescence-activated cell sorting (FACS) using a Sony MA900 cell sorter with a 100 μm nozzle to ensure gentle handling of viable cells. RFP-positive CRC cells were gated and sorted directly into RNAse-free microtubes. Total RNA was extracted from sorted cells using the Maxwell® RSC SimplyRNA Tissue Kit (Promega), following the manufacturer’s protocol. RNA quality and concentration were assessed by spectrophotometry (Nanodrop 2000, Thermo Fisher). Reverse transcription of 500 ng RNA per sample was performed using the GoScript™ Reverse Transcription Mix, Random Primers (Promega) to generate complementary DNA (cDNA).

Candidate genes for quantitative real-time PCR (qRT-PCR) were selected that showed upregulation in liver metastases compared to primary tumors and which showed higher expression in liver epithelium compared to colon epithelium (LM/LE adaptation genes) or which showed the opposite pattern, i.e. higher expression in primary tumor compared to liver metastasis and higher expression in colon compared to liver epithelial cells (PT/CE genes) across patients. qRT-PCR was carried out using the GoTaq® qPCR Master Mix (Promega) on a CFX96 Touch Real-Time PCR Detection System (Bio-Rad). Reactions were performed in technical triplicates using validated primer sets specific to the candidate genes of interest and the housekeeping gene HPRT1 (Table S9). Relative gene expression was calculated using the ΔΔCt method, normalized to monoculture controls.

For statistical assessment of the qPCR validation experiments, *p*-values were calculated on the biological replicate level using ΔCt values and paired two-sided t-tests relative to the NCM460 reference condition.

### Quantification and statistical analysis

#### Raw data analysis and quality control

Raw single cell data were first analyzed with the *Cell Ranger* pipeline from 10x Genomics.^111^ The filtered feature matrices were read in to analyze the data using Seurat (version 4.0.3),^112^^,^^113^^,^^114^^,^^115^ excluding genes that were expressed in less than three cells and excluding cells that expressed less than 1000 different genes. The quality was assessed for each sample separately, excluding cells that had a higher number of detected genes or UMIs than a respective threshold, defined by either three median absolute deviations from the median or three standard deviations from the mean, choosing the more stringent of the two for every individual cell. Initially, all cells with a fraction of mitochondrial genes (*percMito*) higher than 30% regarding total UMIs were excluded. After preliminary clustering the top 15% of cells regarding the percMito were additionally excluded due to a cell type dependency, allowing an individual cell type specific threshold for this parameter, thus taking physiological differences between cell types into account.^116^

#### UMAP projection and cell type annotation of scRNA-seq

The filtered data from above was further normalized for UMAP projection, clustering and cell type annotation. Samples that were sequenced in the same run were merged, normalize (*Seurat::NormalizeData* with *normalization.method=‘LogNormalize’* and scaled (e.g. the overall expression of a gene among all cells was transformed to a mean expression of 0 with a variance of 1), according to the workflow provided by the Satija Lab.^117^ The samples were integrated for the UMAP projection using Canonical Correlation Analysis to match similar cell types from different batches (built-in Seurat method^112^). After identifying the most variable features (*Seurat::FindVariableFeatures* with *selection.method=’vst’, nfeatures=6000, assay=‘RNA’*) principal component analysis (PCA) was performed based on these genes. Cells were clustered following the analysis pipeline of the developers of Seurat^114^ and Luecken & Theis,^118^ using the clustering algorithm of the Seurat platform, including a MMN-graph and Louvain algorithm. For that, a K-nearest neighbor graph based on Euclidean distance in the space defined by PCA and the number of principal components was constructed, with edge weights based on Jaccard similarity (i.e. being neighbors in space).^119^ The Louvain algorithm was then used to iteratively group cells together with the goal of optimizing the standard modularity function.^120^ The Uniform Manifold Approximation and Projection (UMAP) algorithm^121^^,^^122^ was used to generate a non-linear visualization of the data.

Differential expression analysis to find top marker genes for each cluster was performed using the previously defined most variable features, the algorithm MAST,^123^ a minimum fraction of cells expressing the differentially expressed genes of 0.4 and a minimum log-fold change in expression of 0.1. Cell types were defined using common surface markers and top differentially expressed genes per cluster with the help of PanglaoDB,^124^ a public database of a variety of scRNA-seq experiments which allows to browse gene expression and cell type annotations from published data sets. EPCAM was used to identify epithelial cells. Among EPCAM-positive cells, cells of cancer origin were discriminated using inferCNV^125^ (settings as recommended by the developers). For immune cells, CD79A was used to identify B cell species and NKG7, TRBC2 and IL7R for T cells. PDGFRA, collagen species and matrix-specific genes discriminated against fibroblast species. VWF was used as a marker of endothelial cells.

#### Defining differentially expressed genes between cell type groups

We developed a pipeline to estimate differential expression changes, which delivers stable and unbiased results even if there are big differences in the sequencing depth between the groups of cells we are aiming to compare (Figures 2A and S3A).

Starting with the filtered but not normalized or integrated expression values, cells were divided into cell groups based on their cell type (primary tumor cells, liver metastasis cells, colon epithelial cells and liver epithelial cells) and donor they originate from. As comparisons are performed within batch, no batch correction is necessary for this analysis. Cell groups containing less than 49 cells were removed from the analysis. Next, all genes which were not expressed in at least 10% of the cells per cell group contained in the analysis are removed (6872 genes remained in the analysis). Since genes with many zeros were removed, we assumed that the vast majority of the remaining genes are actually expressed and hence only consider gene expression >0 into account, allowing us to better account for big differences in the sequencing depth. Therefore, after log2 transforming the data, the mean expression of every cell group was estimated, without taking the zeros into account. Subsequently, the mean expressions per cell group were centered, setting the library size of every cell group to 10,000. Log2 fold changes (log2 FC) were estimated donor-wise, using the mean expressions per cell group, always pairing the donor matching cell groups to each other, resulting in donor-specific normalized log2 FC for donor 1 and donor 5. Genes with a normalized log2 FC greater than 0.1 or lower than -0.1, which agree in directionality of the log2 FC in all donor-specific groups are defined as candidate genes. 530/6872 genes were defined to be primary tumor specific, while 305/6872 were metastasis specific.

The approach was additionally utilized to compare not only metastasis and primary tumor but also liver epithelial cells with colon epithelial cells. Cell groups of all four cell types were defined for donor 1 and donor 5, except for the cell group of liver epithelial cells of donor 5, since there were none sequenced. This group was subsidized with epithelial liver cells of donor 3. 5019 genes remained in the analysis after removing genes which were too lowly expressed (<10%) in at least one of the cell groups. Since the gene filtering in the bioinformatic pipeline is influenced by tissues used in the individual analysis, the genes used for defining PT and MT candidates (Figures 2A and 2B) slightly differ from those included in PTCE and MTLE analysis (Figures 3B and 3C). This influences the pseudo-bulk expressions and therefore result in minimal different log2 fold changes estimated between metastasis and primary tumor cells (Figure S4). Due to those differences the PTCE and MTLE candidates are not just a subset of the PT and MT candidates even though there is a high overlap. Again, genes with a normalized log2 FC greater than 0.1 or lower than -0.1, which agree in directionality of the log2 FC in all donor-specific groups are defined as candidate genes.

#### Defining tissue exclusively expressed genes between cell groups

When defining differentially expressed genes, genes which show an expression below 10% in any of the defined cell groups are excluded, leading to the analysis overseeing potentially very interesting genes; genes which are not at all or almost not at all expressed in one cell group but highly expressed in the one we want to compare it to. Therefore, we additionally defined exclusively expressed genes based on the same cell groups used when inferring differentially expressed genes. For each gene the percentage of times it was expressed in each cell group was estimated and subsequently the weighted mean over the cell groups from the same cell types was retrieved. Genes expressed in under 10% of the genes in one cell type and over 20% in the other cell type are defined to be expressed exclusively.

#### FGSEA pathway enrichment on cell type groups

Pathway enrichment was performed utilizing fast gene set enrichment analysis (FGSEA) in the form of the R package *fgsea* (version 1.31.0),^80^ setting the minimum node size to 10. Canonical pathways^126^ (accessed on 03.01.2023) from BioCarta, KEGG, PID and Reactome were provided to the algorithm. FGSEA was applied on the log fold changes estimated for each donor between the different cell groups (metastatic cells vs. primary tumor cells & liver epithelial cells vs. colon epithelial cells). Pathways were defined as primary tumor & colon epithelial or liver metastasis & liver epithelial specific based on identical directionality of the NES between tissue comparisons and donors. If a pathway has positive NES across all tissue comparisons and donors, this pathway is defined as MTLE specific. If a pathway has negative NES across all tissue comparisons and donors, it is defined as PTCE specific . These specific pathways were clustered by using k-means^127^ after estimating a distance matrix based on the number of genes pathways share (Jaccard index)^128^ and performing Kruskal's Non-metric Multidimensional Scaling^129^ (*MASS:isoMDS*, package version 7.3-61)^130^ and additionally performed a Fisher test on the genes in Figure 3C as well as on the pathways agreeing in directionality between tissues and donors.

#### Differential expression analysis in external scRNA-seq data

Public available external scRNA-seq data from matching primary colon tumor and liver metastasis was downloaded (*n* = 10, preprocessing and cell type annotation as provided by the authors, GEO accession: GSM7058755).^34^ Differential expression analysis was performed with the same pipeline implemented for our scRNA-seq data. Cells were divided into cell groups based on their cell type and donor they originate from and containing less than 120 cells were removed from the analysis. Next, all genes which were not expressed in at least 5% of the cells per cell group contained in the analysis are removed (6652 genes remained in the analysis). The data was log2 transformed and mean expression of every cell group was estimated (without taking the zeros into account) and centered (setting the library size of every cell group to 10,000). Mean pseudo-bulk metastases and primary tumor expression values were estimated and subsequently the overall mean log2 fold change (MT/PT) was estimated. Pathway enrichment was performed on the log2 fold changes by applying FGSEA, using the same set of pathways we enriched in our scRNA-seq data.

#### Differential expression analysis in external microarray data

Microarray data from two primary colon tumor data sets and one liver metastasis data set was preprocessed together (GEO accession: GSE79959, GSE96528, GSE79959).^63^^,^^64^^,^^65^^,^^66^^,^^67^ Datasets with over 150 samples were subsampled to a maximum of 150 samples per dataset (Table S8) and subsequently preprocessed together via robust multi-average normalization using the *oligo* R package (version 1.68.1).^131^ Principal component analysis (PCA) revealed an overlap of the primary tumor samples in the first and second PC while the samples of the liver metastasis separated from them (Figure S5B). Differential expression analysis between the metastasis and primary tumor was performed using the pipeline implemented by *limma* (version: 3.58.1),^132^ resulting in estimated log2 fold changes and adjusted *p*-values. Pathway enrichment was performed on the log2 fold changes by applying FGSEA, using the same set of pathways we enriched in our scRNA-seq data. Pathway activity was estimated on the pseudo-bulk log2 expression values per cell type per donor using single samples GSEA^133^ implemented in the R package GSVA^134^ (version 2.2.1).

#### Differential expression of co-cultured cell lines by quantitative PCR

The averages of three independent experiments with three technical replicates each were used for statistical analysis (SEM). Two-tailed t-test were applied to test for differences between every liver-like cell line vs the colon-like cell line individually. Asterisks in Figure S6 indicate *p*-values <0.05.

## Supplemental information

Document S1. Figures S1–S7

Table S1. Human donor and sample information

Table S2. Overview of all cells after quality control Cell-specific QC values, sample and cell type information, as well as UMAP projection are provided.

Table S3. DE comparison of liver metastasis vs. primary colon tumor cells (PT & MT specific analysis) All genes included in the analysis are provided. Log2 FC of the individual donors, the mean log2 FC over all donors and a binary classification if a gene is metastasis- or primary tumor-specific are provided for every gene. Log2 FC of the external scRNA-seq data and log2 FC and adjusted *p* value of external microarray data analysis is included.

Table S4. Overview of ‘tissue-exclusive’ genes Percent of cells in which the gene is expressed per cell type is provided, and a binary classification if a gene is metastasis vs. primary tumor specific or metastasis vs. primary tumor AND liver vs. colon epithelial specific is provided for every gene. Log2 FC and adjusted *p* value of external microarray data analysis are included.

Table S5. Pathway enrichment analysis compares liver metastasis vs. primary colon tumor or liver vs. colon epithelial cells (PT & MT, CE & LE specific analysis) Normalized enrichment scores and adjusted *p* values of the individual donors as well as the mean over all donors and a binary classification if a gene is metastasis vs. primary tumor specific or liver vs. colon epithelial specific are provided for every pathway.

Table S6. DE comparison of liver metastasis vs. primary colon tumor and liver vs. colon epithelial cells (PTCE & MTLE specific analysis) Log2 FC of the individual donors, the mean over all donors, and a binary classification if a gene is metastasis vs. primary tumor specific, liver vs. colon epithelial specific, or specific for both are provided for every gene. Log2 FC of the external scRNA-seq data and log2 FC and adjusted *p* value of external microarray data analysis are included. All genes included in the analysis are provided.

Table S7. Pathway enrichment analysis to identify tissue-adaptive pathways comparing liver metastasis vs. primary colon tumor and liver vs. colon epithelial cells (PTCE & MTLE specific analysis) Normalized enrichment scores and adjusted *p* values of the individual donors as well as the mean over all donors and a binary classification if a gene is metastasis vs. primary tumor specific or metastasis vs. primary tumor AND liver vs. colon epithelial specific are provided for every pathway. Clusters of pathways are provided. Normalized enrichment scores and adjusted *p* values of pathway enrichment of the external scRNA-seq data and external microarray data analysis are included. All pathways included in the analysis are provided.

Table S8. List of microarray samples used from the external microarray datasets (see Methods)

Table S9. qPCR primers to investigate candidate adaptive genes from co-culture experiments

## References

[1] Fares J., Fares M.Y., Khachfe H.H., Salhab H.A., Fares Y. (2020). Molecular principles of metastasis: a hallmark of cancer revisited. Signal Transduct. Target. Ther..

[2] Massagué J., Obenauf A.C. (2016). Metastatic colonization by circulating tumour cells. Nature.

[3] Chambers A.F., Groom A.C., MacDonald I.C. (2002). Dissemination and growth of cancer cells in metastatic sites. Nat. Rev. Cancer.

[4] Luzzi K.J., MacDonald I.C., Schmidt E.E., Kerkvliet N., Morris V.L., Chambers A.F., Groom A.C. (1998). Multistep Nature of Metastatic Inefficiency: Dormancy of Solitary Cells after Successful Extravasation and Limited Survival of Early Micrometastases. Am. J. Pathol..

[5] Gupta G.P., Massagué J. (2006). Cancer metastasis: building a framework. Cell.

[6] Weiss L. (1990). Metastatic Inefficiency. Adv. Cancer Res..

[7] Sugarbaker P.H. (1993). Metastatic inefficiency: The scientific basis for resection of liver metastases from colorectal cancer. J. Surg. Oncol..

[8] Celià-Terrassa T., Kang Y. (2016). Distinctive properties of metastasis-initiating cells. Genes Dev..

[9] Steeg P.S. (2006). Tumor metastasis: mechanistic insights and clinical challenges. Nat. Med..

[10] Paget S. (1989). The distribution of secondary growths in cancer of the breast. 1889. Cancer Metastasis Rev..

[11] Abrantes A.M., Caetano-Oliveira R., Oliveiros B., Cipriano M.A., Tralhão J.G. (2025). Association Between Colorectal Cancer Primary Features and Liver Metastases Histological Growth Patterns: Inflammation on the Primary Tumor is Associated with Desmoplastic Growth Pattern. Clin. Colorectal Cancer.

[12] Ewing J. (1922).

[13] Weigelt B., Glas A.M., Wessels L.F.A., Witteveen A.T., Peterse J.L., van't Veer L.J. (2003). Gene expression profiles of primary breast tumors maintained in distant metastases. Proc. Natl. Acad. Sci..

[14] Budczies J., von Winterfeld M., Klauschen F., Bockmayr M., Lennerz J.K., Denkert C., Wolf T., Warth A., Dietel M., Anagnostopoulos I. (2015). The landscape of metastatic progression patterns across major human cancers. Oncotarget.

[15] Riker A.I., Enkemann S.A., Fodstad O., Liu S., Ren S., Morris C., Xi Y., Howell P., Metge B., Samant R.S. (2008). The gene expression profiles of primary and metastatic melanoma yields a transition point of tumor progression and metastasis. BMC Med. Genomics.

[16] Tang Z., Li C., Kang B., Gao G., Li C., Zhang Z. (2017). GEPIA: a web server for cancer and normal gene expression profiling and interactive analyses. Nucleic Acids Res..

[17] Baffa R., Fassan M., Volinia S., O'Hara B., Liu C.G., Palazzo J.P., Gardiman M., Rugge M., Gomella L.G., Croce C.M., Rosenberg A. (2009). MicroRNA expression profiling of human metastatic cancers identifies cancer gene targets. J. Pathol..

[18] Wang R., Li J., Zhou X., Mao Y., Wang W., Gao S., Wang W., Gao Y., Chen K., Yu S. (2022). Single-cell genomic and transcriptomic landscapes of primary and metastatic colorectal cancer tumors. Genome Med..

[19] Brooks S.A., Lomax-Browne H.J., Carter T.M., Kinch C.E., Hall D.M.S. (2010). Molecular interactions in cancer cell metastasis. Acta Histochem..

[20] Brabletz T. (2012). To differentiate or not — routes towards metastasis. Nat. Rev. Cancer.

[21] Geiger T.R., Peeper D.S. (2009). Metastasis mechanisms. Biochim. Biophys. Acta.

[22] Simeonov K.P., Byrns C.N., Clark M.L., Norgard R.J., Martin B., Stanger B.Z., Shendure J., McKenna A., Lengner C.J. (2021). Single-cell lineage tracing of metastatic cancer reveals selection of hybrid EMT states. Cancer Cell.

[23] Garner H., de Visser K.E. (2020). Immune crosstalk in cancer progression and metastatic spread: a complex conversation. Nat. Rev. Immunol..

[24] Blomberg O.S., Spagnuolo L., de Visser K.E. (2018). Immune regulation of metastasis: mechanistic insights and therapeutic opportunities. Dis. Model. Mech..

[25] Liu J., Cho Y.B., Hong H.K., Wu S., Ebert P.J., Bray S.M., Wong S.S., Ting J.C., Calley J.N., Whittington C.F. (2020). Molecular dissection of CRC primary tumors and their matched liver metastases reveals critical role of immune microenvironment, EMT and angiogenesis in cancer metastasis. Sci. Rep..

[26] Schild T., Low V., Blenis J., Gomes A.P. (2018). Unique Metabolic Adaptations Dictate Distal Organ-Specific Metastatic Colonization. Cancer Cell.

[27] Jia D., Park J.H., Jung K.H., Levine H., Kaipparettu B.A. (2018). Elucidating the Metabolic Plasticity of Cancer: Mitochondrial Reprogramming and Hybrid Metabolic States. Cells.

[28] Obenauf A.C., Massagué J. (2015). Surviving at a Distance: Organ-Specific Metastasis. Trends Cancer.

[29] Borrelli C., Roberts M., Eletto D., Hussherr M.D., Fazilaty H., Valenta T., Lafzi A., Kretz J.A., Guido Vinzoni E., Karakatsani A. (2024). In vivo interaction screening reveals liver-derived constraints to metastasis. Nature.

[30] Cioce M., Fumagalli M.R., Donzelli S., Goeman F., Canu V., Rutigliano D., Orlandi G., Sacconi A., Pulito C., Palcau A.C. (2023). Interrogating colorectal cancer metastasis to liver: a search for clinically viable compounds and mechanistic insights in colorectal cancer Patient Derived Organoids. J. Exp. Clin. Cancer Res..

[31] Cheng J., Song X., Ao L., Chen R., Chi M., Guo Y., Zhang J., Li H., Zhao W., Guo Z., Wang X. (2018). Shared liver-like transcriptional characteristics in liver metastases and corresponding primary colorectal tumors. J. Cancer.

[32] Teng S., Li Y.E., Yang M., Qi R., Huang Y., Wang Q., Zhang Y., Chen S., Li S., Lin K. (2020). Tissue-specific transcription reprogramming promotes liver metastasis of colorectal cancer. Cell Res..

[33] Li S., Yang M., Teng S., Lin K., Wang Y., Zhang Y., Guo W., Wang D. (2023). Chromatin accessibility dynamics in colorectal cancer liver metastasis: Uncovering the liver tropism at single cell resolution. Pharmacol. Res..

[34] Wang F., Long J., Li L., Wu Z.X., Da T.T., Wang X.Q., Huang C., Jiang Y.H., Yao X.Q., Ma H.Q. (2023). Single-cell and spatial transcriptome analysis reveals the cellular heterogeneity of liver metastatic colorectal cancer. Sci. Adv..

[35] Che L.-H., Liu J.W., Huo J.P., Luo R., Xu R.M., He C., Li Y.Q., Zhou A.J., Huang P., Chen Y.Y. (2021). A single-cell atlas of liver metastases of colorectal cancer reveals reprogramming of the tumor microenvironment in response to preoperative chemotherapy. Cell Discov..

[36] Basnet H., Tian L., Ganesh K., Huang Y.H., Macalinao D.G., Brogi E., Finley L.W., Massagué J. (2019). Flura-seq identifies organ-specific metabolic adaptations during early metastatic colonization. eLife.

[37] De Jong M.C., Pulitano C., Ribero D., Strub J., Mentha G., Schulick R.D., Choti M.A., Aldrighetti L., Capussotti L., Pawlik T.M. (2009). Rates and Patterns of Recurrence Following Curative Intent Surgery for Colorectal Liver Metastasis: An International Multi-Institutional Analysis of 1669 Patients. Ann. Surg..

[38] Scherman P., Syk I., Holmberg E., Naredi P., Rizell M. (2021). Impact of patient, primary tumor and metastatic pattern including tumor location on survival in patients undergoing ablation or resection for colorectal liver metastases: A population-based national cohort study. Eur. J. Surg. Oncol..

[39] Zhang L., Zhou R., Zhang W., Yao X., Li W., Xu L., Sun X., Zhao L. (2019). Cysteine-rich intestinal protein 1 suppresses apoptosis and chemosensitivity to 5-fluorouracil in colorectal cancer through ubiquitin-mediated Fas degradation. J. Exp. Clin. Cancer Res..

[40] Zhang L.-Z., Huang L.-Y., Huang A.-L., Liu J.-X., Yang F. (2018). CRIP1 promotes cell migration, invasion and epithelial-mesenchymal transition of cervical cancer by activating the Wnt/β-catenin signaling pathway. Life Sci..

[41] Liu Y., Li W., Luo J., Wu Y., Xu Y., Chen T., Zhang W., Fu F. (2021). Cysteine-Rich Intestinal Protein 1 Served as an Epithelial Ovarian Cancer Marker via Promoting Wnt/β-Catenin-Mediated EMT and Tumour Metastasis. Dis. Markers.

[42] Smathers R.L., Petersen D.R. (2011). The human fatty acid-binding protein family: Evolutionary divergences and functions. Hum. Genomics.

[43] Schroeder F., McIntosh A.L., Martin G.G., Huang H., Landrock D., Chung S., Landrock K.K., Dangott L.J., Li S., Kaczocha M. (2016). Fatty Acid Binding Protein-1 (FABP1) and the Human FABP1 T94A Variant: Roles in the Endocannabinoid System and Dyslipidemias. Lipids.

[44] Dum D., Ocokoljic A., Lennartz M., Hube-Magg C., Reiswich V., Höflmayer D., Jacobsen F., Bernreuther C., Lebok P., Sauter G. (2022). FABP1 expression in human tumors: a tissue microarray study on 17,071 tumors. Virchows Arch..

[45] McKillop I.H., Girardi C.A., Thompson K.J. (2019). Role of fatty acid binding proteins (FABPs) in cancer development and progression. Cell. Signal..

[46] Chung Y.-H., Hung T.H., Yu C.F., Tsai C.K., Weng C.C., Jhang F., Chen F.H., Lin G. (2023). Glycolytic Plasticity of Metastatic Lung Cancer Captured by Noninvasive 18F-FDG PET/CT and Serum 1H-NMR Analysis: An Orthotopic Murine Model Study. Metabolites.

[47] Graziano F., Ruzzo A., Giacomini E., Ricciardi T., Aprile G., Loupakis F., Lorenzini P., Ongaro E., Zoratto F., Catalano V. (2017). Glycolysis gene expression analysis and selective metabolic advantage in the clinical progression of colorectal cancer. Pharmacogenomics J..

[48] Lum J.J., Bui T., Gruber M., Gordan J.D., DeBerardinis R.J., Covello K.L., Simon M.C., Thompson C.B. (2007). The transcription factor HIF-1α plays a critical role in the growth factor-dependent regulation of both aerobic and anaerobic glycolysis. Genes Dev..

[49] Rankin E.B., Giaccia A.J. (2016). Hypoxic control of metastasis. Science.

[50] Wang Y., Nakajima T., Gonzalez F.J., Tanaka N. (2020). PPARs as Metabolic Regulators in the Liver: Lessons from Liver-Specific PPAR-Null Mice. Int. J. Mol. Sci..

[51] Ide T., Shimano H., Yoshikawa T., Yahagi N., Amemiya-Kudo M., Matsuzaka T., Nakakuki M., Yatoh S., Iizuka Y., Tomita S. (2003). Cross-Talk between Peroxisome Proliferator-Activated Receptor (PPAR) α and Liver X Receptor (LXR) in Nutritional Regulation of Fatty Acid Metabolism. II. LXRs Suppress Lipid Degradation Gene Promoters through Inhibition of PPAR Signaling. Mol. Endocrinol..

[52] PID_HNF3A_PATHWAY. https://www.gsea-msigdb.org/gsea/msigdb/cards/PID_HNF3A_PATHWAY.

[53] Tomofuji K., Kondo J., Onuma K., Coppo R., Horie H., Oyama K., Miyoshi E., Fukumitsu K., Ishii T., Hatano E., Inoue M. (2023). Hepatocyte differentiation from mouse liver ductal organoids by transducing 4 liver-specific transcription factors. Hepatol. Commun..

[54] BIOCARTA_INSULIN_PATHWAY. https://www.gsea-msigdb.org/gsea/msigdb/human/geneset/BIOCARTA_INSULIN_PATHWAY.html.

[55] Najjar S.M., Perdomo G. (2019). Hepatic Insulin Clearance: Mechanism and Physiology. Physiology.

[56] Saltiel A.R., Kahn C.R. (2001). Insulin signalling and the regulation of glucose and lipid metabolism. Nature.

[57] Gopinathrao G. (2008). Metabolism of polyamines. Reactome.

[58] Nakamura A., Kurihara S., Takahashi D., Ohashi W., Nakamura Y., Kimura S., Onuki M., Kume A., Sasazawa Y., Furusawa Y. (2021). Symbiotic polyamine metabolism regulates epithelial proliferation and macrophage differentiation in the colon. Nat. Commun..

[59] KEGG PATHWAY Propanoate metabolism - Reference pathway. https://www.kegg.jp/pathway/map00640.

[60] Louis P., Flint H.J. (2017). Formation of propionate and butyrate by the human colonic microbiota. Environ. Microbiol..

[61] Martínez-Ruiz M., Robeson M.S., Piccolo B.D. (2025). Fueling the fire: colonocyte metabolism and its effect on the colonic epithelia. Crit. Rev. Food Sci. Nutr..

[62] Carretta M.D., Quiroga J., López R., Hidalgo M.A., Burgos R.A. (2021). Participation of Short-Chain Fatty Acids and Their Receptors in Gut Inflammation and Colon Cancer. Front. Physiol..

[63] Sveen A., Johannessen B., Tengs T., Danielsen S.A., Eilertsen I.A., Lind G.E., Berg K.C.G., Leithe E., Meza-Zepeda L.A., Domingo E. (2017). Multilevel genomics of colorectal cancers with microsatellite instability-clinical impact of JAK1 mutations and consensus molecular subtype 1. Genome Med..

[64] Sveen A., Bruun J., Eide P.W., Eilertsen I.A., Ramirez L., Murumägi A., Arjama M., Danielsen S.A., Kryeziu K., Elez E. (2018). Colorectal Cancer Consensus Molecular Subtypes Translated to Preclinical Models Uncover Potentially Targetable Cancer Cell Dependencies. Clin. Cancer Res..

[65] Eide P.W., Moosavi S.H., Eilertsen I.A., Brunsell T.H., Langerud J., Berg K.C.G., Røsok B.I., Bjørnbeth B.A., Nesbakken A., Lothe R.A., Sveen A. (2021). Metastatic heterogeneity of the consensus molecular subtypes of colorectal cancer. npj Genom. Med..

[66] Bruun J., Sveen A., Barros R., Eide P.W., Eilertsen I., Kolberg M., Pellinen T., David L., Svindland A., Kallioniemi O. (2018). Prognostic, predictive, and pharmacogenomic assessments of CDX2 refine stratification of colorectal cancer. Mol. Oncol..

[67] Smeby J., Sveen A., Bergsland C.H., Eilertsen I.A., Danielsen S.A., Eide P.W., Hektoen M., Guren M.G., Nesbakken A., Bruun J., Lothe R.A. (2019). Exploratory analyses of consensus molecular subtype-dependent associations of TP53 mutations with immunomodulation and prognosis in colorectal cancer. ESMO Open.

[68] Wang G., Bonkovsky H.L., de Lemos A., Burczynski F.J. (2015). Recent insights into the biological functions of liver fatty acid binding protein 1. J. Lipid Res..

[69] Raabe M., Véniant M.M., Sullivan M.A., Zlot C.H., Björkegren J., Nielsen L.B., Wong J.S., Hamilton R.L., Young S.G. (1999). Analysis of the role of microsomal triglyceride transfer protein in the liver of tissue-specific knockout mice. J. Clin. Investig..

[70] Wice B.M., Gordon J.I. (1995). A tetraspan membrane glycoprotein produced in the human intestinal epithelium and liver that can regulate cell density-dependent proliferation. J. Biol. Chem..

[71] Schueler M., Braun D.A., Chandrasekar G., Gee H.Y., Klasson T.D., Halbritter J., Bieder A., Porath J.D., Airik R., Zhou W. (2015). DCDC2 mutations cause a renal-hepatic ciliopathy by disrupting Wnt signaling. Am. J. Hum. Genet..

[72] Grammatikopoulos T., Sambrotta M., Strautnieks S., Foskett P., Knisely A.S., Wagner B., Deheragoda M., Starling C., Mieli-Vergani G., Smith J. (2016). Mutations in DCDC2 (doublecortin domain containing protein 2) in neonatal sclerosing cholangitis. J. Hepatol..

[73] Rouillard A.D., Gundersen G.W., Fernandez N.F., Wang Z., Monteiro C.D., McDermott M.G., Ma'ayan A. (2016). The harmonizome: a collection of processed datasets gathered to serve and mine knowledge about genes and proteins. Database..

[74] Diamant I., Clarke D.J.B., Evangelista J.E., Lingam N., Ma’ayan A. (2025). Harmonizome 3.0: integrated knowledge about genes and proteins from diverse multi-omics resources. Nucleic Acids Res..

[75] Kanehisa M. (2019). Toward understanding the origin and evolution of cellular organisms. Protein Sci..

[76] Kanehisa M., Goto S. (2000). KEGG: Kyoto Encyclopedia of Genes and Genomes. Nucleic Acids Res..

[77] Schaefer C.F., Anthony K., Krupa S., Buchoff J., Day M., Hannay T., Buetow K.H. (2009). PID: the Pathway Interaction Database. Nucleic Acids Res..

[78] Jassal B., Matthews L., Viteri G., Gong C., Lorente P., Fabregat A., Sidiropoulos K., Cook J., Gillespie M., Haw R. (2020). The reactome pathway knowledgebase. Nucleic Acids Res..

[79] Milacic M., Beavers D., Conley P., Gong C., Gillespie M., Griss J., Haw R., Jassal B., Matthews L., May B. (2024). The Reactome Pathway Knowledgebase 2024. Nucleic Acids Res..

[80] Korotkevich G., Sukhov V., Budin N., Shpak B., Artyomov M.N., Sergushichev A. (2016). Fast Gene Set Enrichment Analysis. bioRxiv.

[81] PID_ARF6_PATHWAY. https://www.gsea-msigdb.org/gsea/msigdb/human/geneset/PID_ARF6_PATHWAY.html?ex=1.

[82] Suzuki T., Kanai Y., Hara T., Sasaki J., Sasaki T., Kohara M., Maehama T., Taya C., Shitara H., Yonekawa H. (2006). Crucial role of the small GTPase ARF6 in hepatic cord formation during liver development. Mol. Cell Biol..

[83] Reactome | Heparan sulfate/heparin (HS-GAG) metabolism. https://reactome.org/content/detail/R-HSA-1638091.

[84] Baghy K., Tátrai P., Regős E., Kovalszky I. (2016). Proteoglycans in liver cancer. World J. Gastroenterol..

[85] Tóth G., Pál D., Sugár S., Kovalszky I., Dezső K., Schlosser G., Drahos L., Turiák L. (2022). Expression of glycosaminoglycans in cirrhotic liver and hepatocellular carcinoma-a pilot study including etiology. Anal. Bioanal. Chem..

[86] Roskams T., Moshage H., De Vos R., Guido D., Yap P., Desmet V. (1995). Heparan sulfate proteoglycan expression in normal human liver. Hepatology.

[87] PID_RHOA_REG_PATHWAY. https://www.gsea-msigdb.org/gsea/msigdb/human/geneset/PID_RHOA_REG_PATHWAY.html.

[88] Reactome | RHOB GTPase cycle. https://reactome.org/content/detail/R-HSA-9013026.

[89] Reactome | Regulation of lipid metabolism by PPARalpha. https://reactome.org/content/detail/R-HSA-400206.

[90] Pang T., Kaufman A., Choi J., Gill A., Drummond M., Hugh T., Samra J. (2015). Peroxisome proliferator-activated receptor-α staining is associated with worse outcome in colorectal liver metastases. Mol. Clin. Oncol..

[91] Morinishi T., Tokuhara Y., Ohsaki H., Ibuki E., Kadota K., Hirakawa E. (2019). Activation and Expression of Peroxisome Proliferator-Activated Receptor Alpha Are Associated with Tumorigenesis in Colorectal Carcinoma. PPAR Res..

[92] Buendia I., Michalska P., Navarro E., Gameiro I., Egea J., León R. (2016). Nrf2–ARE pathway: An emerging target against oxidative stress and neuroinflammation in neurodegenerative diseases. Pharmacol. Ther..

[93] Bourgonje A.R., Kloska D., Grochot-Przęczek A., Feelisch M., Cuadrado A., van Goor H. (2023). Personalized redox medicine in inflammatory bowel diseases: an emerging role for HIF-1α and NRF2 as therapeutic targets. Redox Biol..

[94] KEGG_OXIDATIVE_PHOSPHORYLATION. https://www.gsea-msigdb.org/gsea/msigdb/cards/KEGG_OXIDATIVE_PHOSPHORYLATION.

[95] Boyer P.D. (1997). The ATP synthase—a splendid molecular machine. Annu. Rev. Biochem..

[96] Welker A.F., Moreira D.C., Campos É.G., Hermes-Lima M. (2013). Role of redox metabolism for adaptation of aquatic animals to drastic changes in oxygen availability. Comp. Biochem. Physiol. Mol. Integr. Physiol..

[97] Barbour J.A., Turner N. (2014). Mitochondrial Stress Signaling Promotes Cellular Adaptations. Int. J. Cell Biol..

[98] Forgac M. (2007). Vacuolar ATPases: rotary proton pumps in physiology and pathophysiology. Nat. Rev. Mol. Cell Biol..

[99] Chen F., Kang R., Liu J., Tang D. (2022). The V-ATPases in cancer and cell death. Cancer Gene Ther..

[100] Boedtkjer E., Pedersen S.F. (2020). The Acidic Tumor Microenvironment as a Driver of Cancer. Annu. Rev. Physiol..

[101] McGuirk S., Audet-Delage Y., St-Pierre J. (2020). Metabolic Fitness and Plasticity in Cancer Progression. Trends Cancer.

[102] Moorman A., Benitez E.K., Cambulli F., Jiang Q., Mahmoud A., Lumish M., Hartner S., Balkaran S., Bermeo J., Asawa S. (2024). Progressive plasticity during colorectal cancer metastasis. Nature.

[103] Jiang Y., Long G., Huang X., Wang W., Cheng B., Pan W. (2025). Single-cell transcriptomic analysis reveals dynamic changes in the liver microenvironment during colorectal cancer metastatic progression. J. Transl. Med..

[104] Househam J., Heide T., Cresswell G.D., Spiteri I., Kimberley C., Zapata L., Lynn C., James C., Mossner M., Fernandez-Mateos J. (2022). Phenotypic plasticity and genetic control in colorectal cancer evolution. Nature.

[105] Fuertes-Agudo M., Luque-Tévar M., Cucarella C., Martín-Sanz P., Casado M. (2023). Advances in Understanding the Role of NRF2 in Liver Pathophysiology and Its Relationship with Hepatic-Specific Cyclooxygenase-2 Expression. Antioxidants.

[106] Hu M., Yuan L., Zhu J. (2024). The Dual Role of NRF2 in Colorectal Cancer: Targeting NRF2 as a Potential Therapeutic Approach. J. Inflamm. Res..

[107] Pouremamali F., Pouremamali A., Dadashpour M., Soozangar N., Jeddi F. (2022). An update of Nrf2 activators and inhibitors in cancer prevention/promotion. Cell Commun. Signal..

[108] Li Y., Pan Y., Zhao X., Wu S., Li F., Wang Y., Liu B., Zhang Y., Gao X., Wang Y., Zhou H. (2024). Peroxisome proliferator-activated receptors: A key link between lipid metabolism and cancer progression. Clin. Nutr..

[109] Zhang Y., Xiao B., Liu Y., Wu S., Xiang Q., Xiao Y., Zhao J., Yuan R., Xie K., Li L. (2024). Roles of PPAR activation in cancer therapeutic resistance: Implications for combination therapy and drug development. Eur. J. Pharmacol..

[110] Krämer M., Plum P.S., Velazquez Camacho O., Folz-Donahue K., Thelen M., Garcia-Marquez I., Wölwer C., Büsker S., Wittig J., Franitza M. (2020). Cell type-specific transcriptomics of esophageal adenocarcinoma as a scalable alternative for single cell transcriptomics. Mol. Oncol..

[111] Zheng G.X.Y., Terry J.M., Belgrader P., Ryvkin P., Bent Z.W., Wilson R., Ziraldo S.B., Wheeler T.D., McDermott G.P., Zhu J. (2017). Massively parallel digital transcriptional profiling of single cells. Nat. Commun..

[112] Butler A., Hoffman P., Smibert P., Papalexi E., Satija R. (2018). Integrating single-cell transcriptomic data across different conditions, technologies, and species. Nat. Biotechnol..

[113] Satija R., Farrell J.A., Gennert D., Schier A.F., Regev A. (2015). Spatial reconstruction of single-cell gene expression data. Nat. Biotechnol..

[114] Hao Y., Hao S., Andersen-Nissen E., Mauck W.M., Zheng S., Butler A., Lee M.J., Wilk A.J., Darby C., Zager M. (2021). Integrated analysis of multimodal single-cell data. Cell.

[115] Stuart T., Butler A., Hoffman P., Hafemeister C., Papalexi E., Mauck W.M., Hao Y., Stoeckius M., Smibert P., Satija R. (2019). Comprehensive Integration of Single-Cell Data. Cell.

[116] Mercer T.R., Neph S., Dinger M.E., Crawford J., Smith M.A., Shearwood A.M.J., Haugen E., Bracken C.P., Rackham O., Stamatoyannopoulos J.A. (2011). The human mitochondrial transcriptome. Cell.

[117] Analysis, visualization, and integration of Visium HD spatial datasets with Seurat. https://satijalab.org/seurat/articles/pbmc3k_tutorial#scaling-the-data.

[118] Luecken M.D., Theis F.J. (2019). Current best practices in single-cell RNA-seq analysis: a tutorial. Mol. Syst. Biol..

[119] Xu C., Su Z. (2015). Identification of cell types from single-cell transcriptomes using a novel clustering method. Bioinformatics.

[120] Blondel V.D., Guillaume J.-L., Lambiotte R., Lefebvre E. (2008). Fast unfolding of communities in large networks. J. Stat. Mech..

[121] McInnes L., Healy J., Melville J.U.M.A.P. (2018). Uniform Manifold Approximation and Projection for Dimension Reduction. arXiv.

[122] Becht E., McInnes L., Healy J., Dutertre C.A., Kwok I.W.H., Ng L.G., Ginhoux F., Newell E.W. (2019). Dimensionality reduction for visualizing single-cell data using UMAP. Nat. Biotechnol..

[123] Finak G., McDavid A., Yajima M., Deng J., Gersuk V., Shalek A.K., Slichter C.K., Miller H.W., McElrath M.J., Prlic M. (2015). MAST: a flexible statistical framework for assessing transcriptional changes and characterizing heterogeneity in single-cell RNA sequencing data. Genome Biol..

[124] Franzén O., Gan L.-M., Björkegren J.L.M. (2019). PanglaoDB: a web server for exploration of mouse and human single-cell RNA sequencing data. Database..

[125] broadinstitute/infercnv (2024). https://github.com/broadinstitute/inferCNV.

[126] GSEA | MSigDB | Human MSigDB Collections https://www.gsea-msigdb.org/gsea/msigdb/human/collections.jsp#C2.

[127] Lloyd S. (1982). Least squares quantization in PCM. IEEE Trans. Inf. Theory.

[128] Murphy A.H. (1996). The Finley Affair: A Signal Event in the History of Forecast Verification. Weather Forecast..

[129] Kruskal J.B. (1964). Nonmetric multidimensional scaling: A numerical method. Psychometrika.

[130] Modern Applied Statistics with S, 4th ed.. https://www.stats.ox.ac.uk/pub/MASS4/.

[131] Carvalho B.S., Irizarry R.A. (2010). A framework for oligonucleotide microarray preprocessing. Bioinformatics.

[132] Ritchie M.E., Phipson B., Wu D., Hu Y., Law C.W., Shi W., Smyth G.K. (2015). limma powers differential expression analyses for RNA-sequencing and microarray studies. Nucleic Acids Res..

[133] Barbie D.A., Tamayo P., Boehm J.S., Kim S.Y., Moody S.E., Dunn I.F., Schinzel A.C., Sandy P., Meylan E., Scholl C. (2009). Systematic RNA interference reveals that oncogenic KRAS-driven cancers require TBK1. Nature.

[134] Hänzelmann S., Castelo R., Guinney J. (2013). GSVA: gene set variation analysis for microarray and RNA-Seq data. BMC Bioinf..

