# Supplementary material for: Gene expression adaptation of metastases to their host tissue
Source: iScience. 2026 Jul 21;29(8):116744. doi: 10.1016/j.isci.2026.116744 (PMC13392873; doi:10.1016/j.isci.2026.116744)
Supplement: Document S1. Figures S1–S7 [file mmc1.pdf]

iScience, Volume *29*

Supplemental information

Gene expression adaptation

# of metastases to their host tissue

Luise Nagel, Marten Wenzel, Sascha Hoppe, Mohammad Karimpour, Patrick S. Plum, Abdossamad Hamoudi, Israt Jahan, Sarah Schmitt, Marek Franitza, Roger Wahba, Marc Bludau, Christiane J. Bruns, Alexander Quaas, Andreas Beyer, and Axel M. Hillmer

Supplementary Data Figures

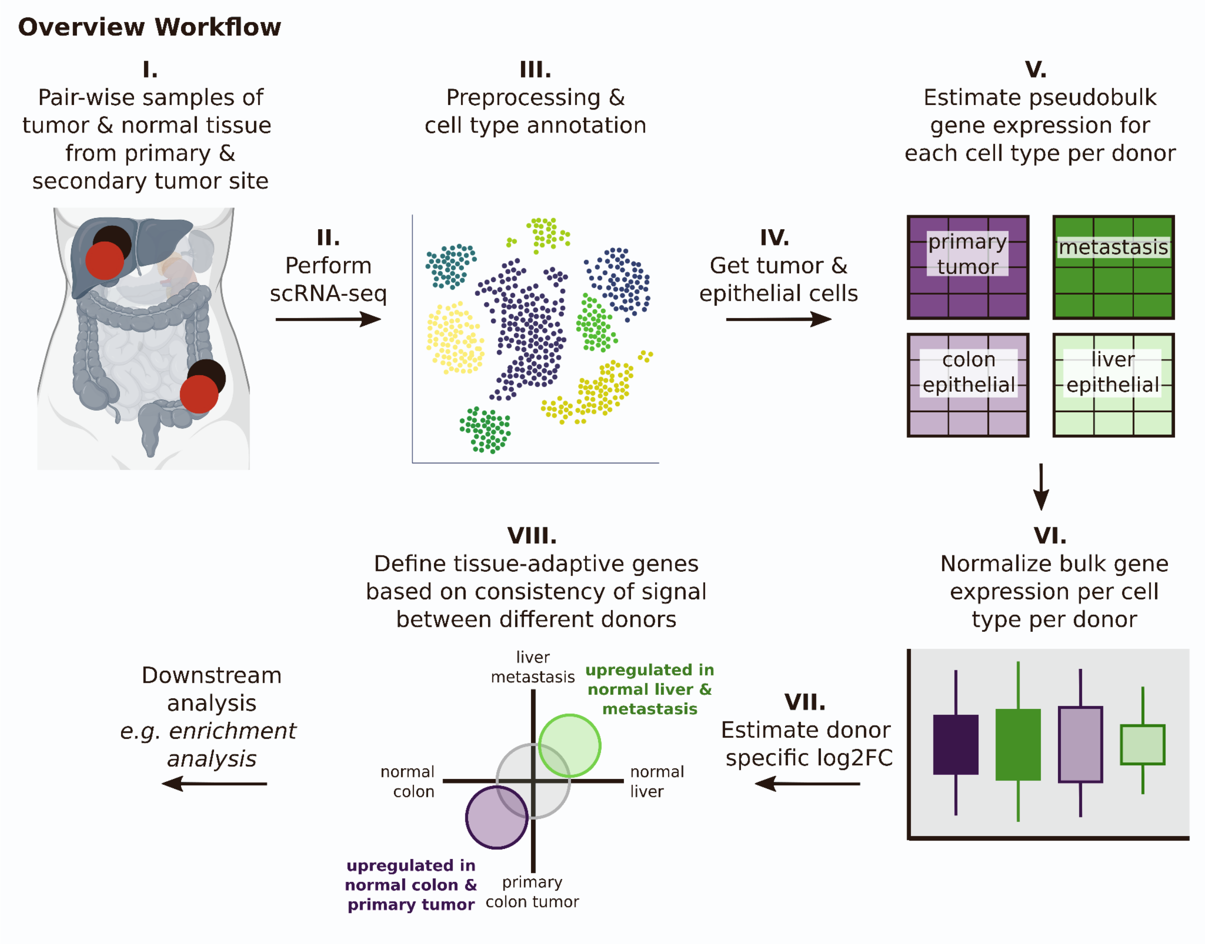

Figure S1: Overview of pipeline to identify tissue-adaptive genes.

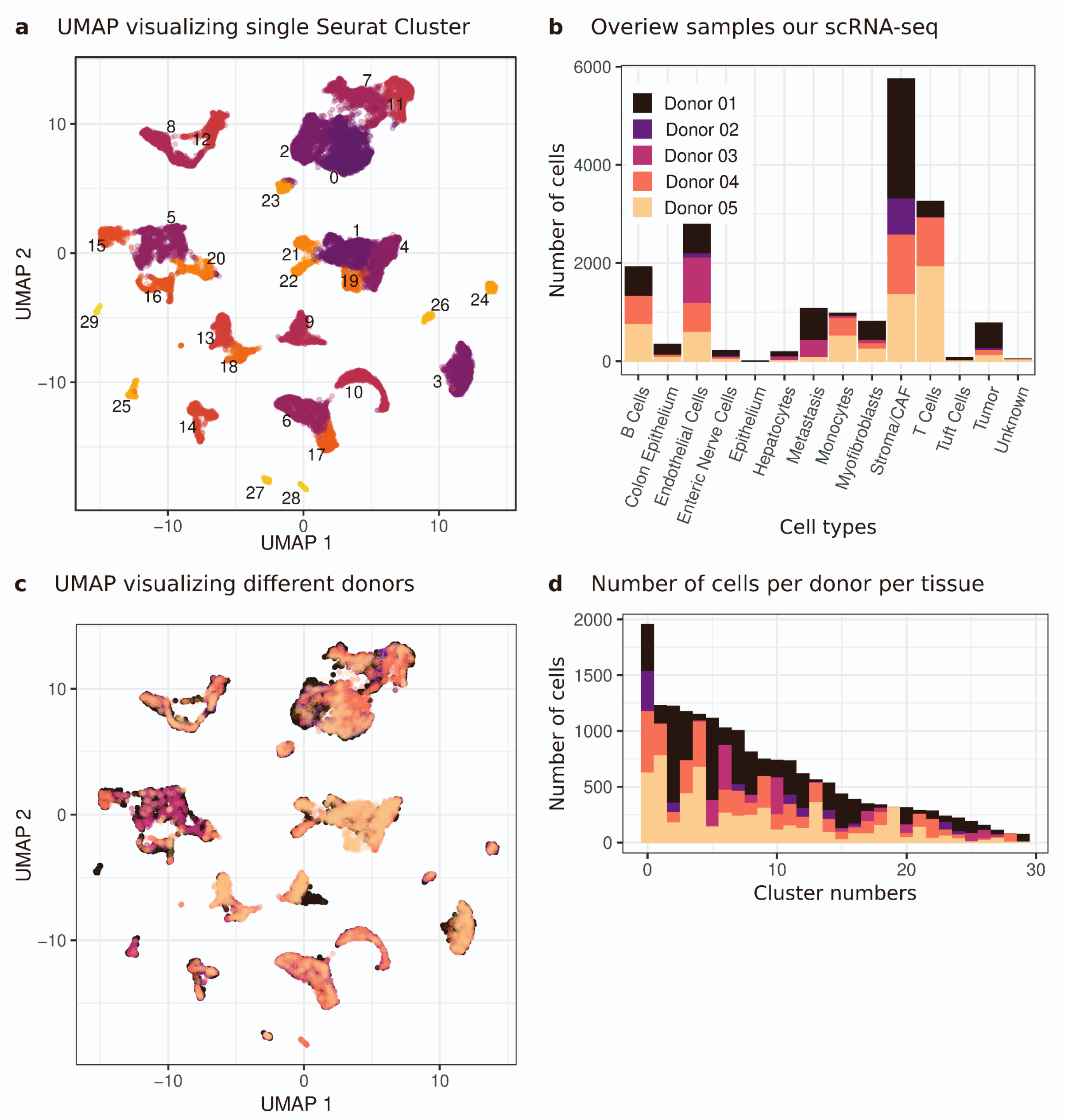

Figure S2: Overview of scRNA-seq dataset from five donors with primary colon tumor and liver metastasis. a) *UMAP of scRNA-seq data with colors indicating different clusters.* ***b)*** *Barplot showing the number of cells per cell type colored by donor.* ***c)*** *UMAP of scRNA-seq data with colors indicating different donor origin/sample site origin.* ***d)*** *Barplot showing the number of cells per cluster per donor.*

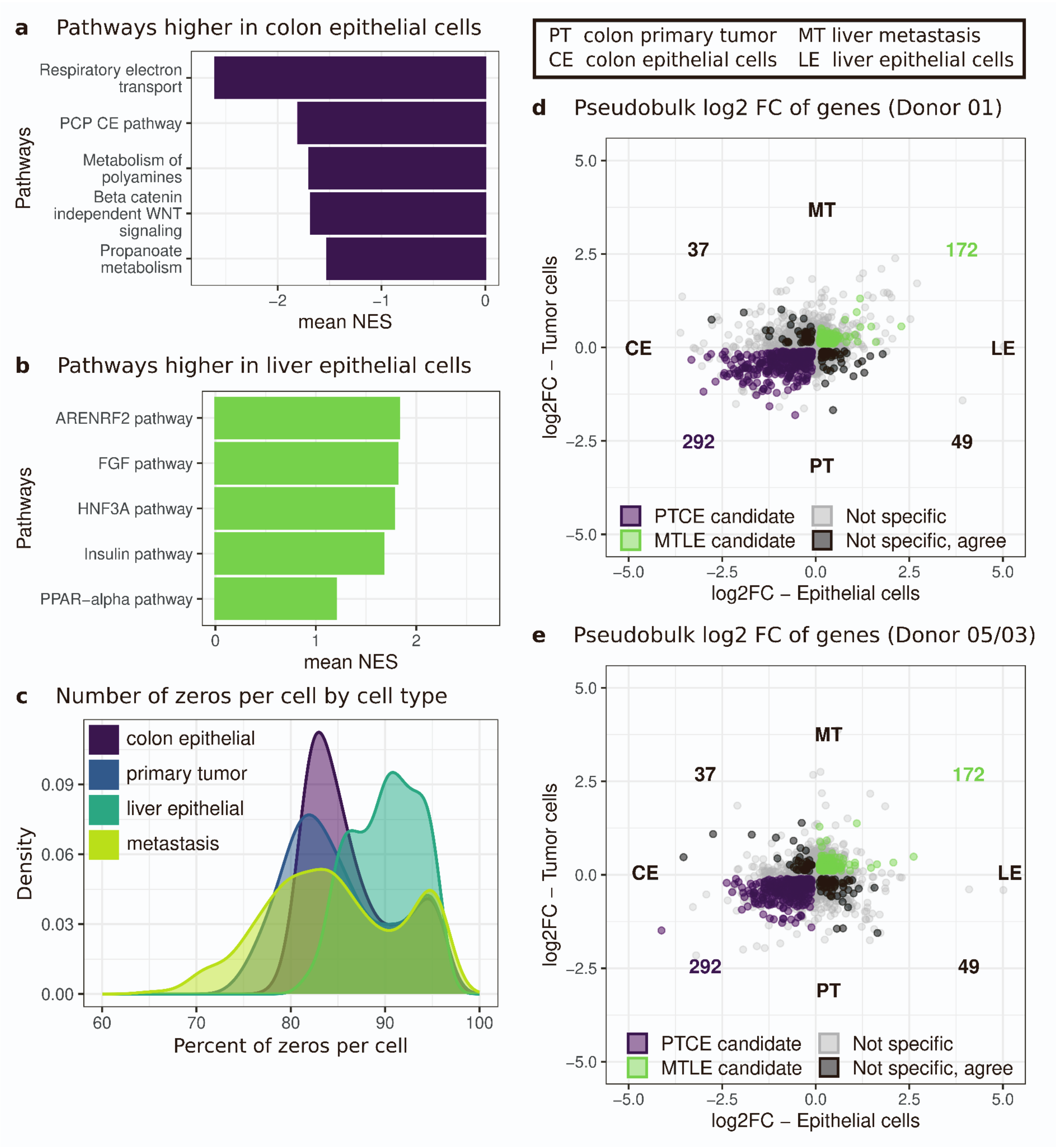

Figure S3: Differences in expression between MT and PT as well as LE and CE cells. a) *Five of the most upregulated pathways in colon epithelial cells when compared to liver epithelial cells. The mean normalized enrichment score (NES) across the donors is displayed.* ***b)*** *Five of the most upregulated pathways in liver epithelial cells compared to colon epithelial cells. Mean NES across the donors is displayed.* ***c)*** *Percent of zeros per cell (after filter for low quality cells, see Methods) for colon epithelial (purple), colon primary tumor (blue), liver epithelial (turquoise) and liver metastatic (light green) cells over all donors.* ***d)*** *Mean log2 fold change of metastasis and primary tumor plotted against the mean log2 fold change of liver and colon epithelial cells of donor 1. Genes with expression patterns consistent between the donors are highlighted (primary tumor and colon epithelial specific genes = purple, metastasis and liver epithelial specific genes = green, other = black, four outlier genes are not visualized in the plot).* ***e)*** *As b) but for donor 5 (liver epithelial cells from donor 3).*

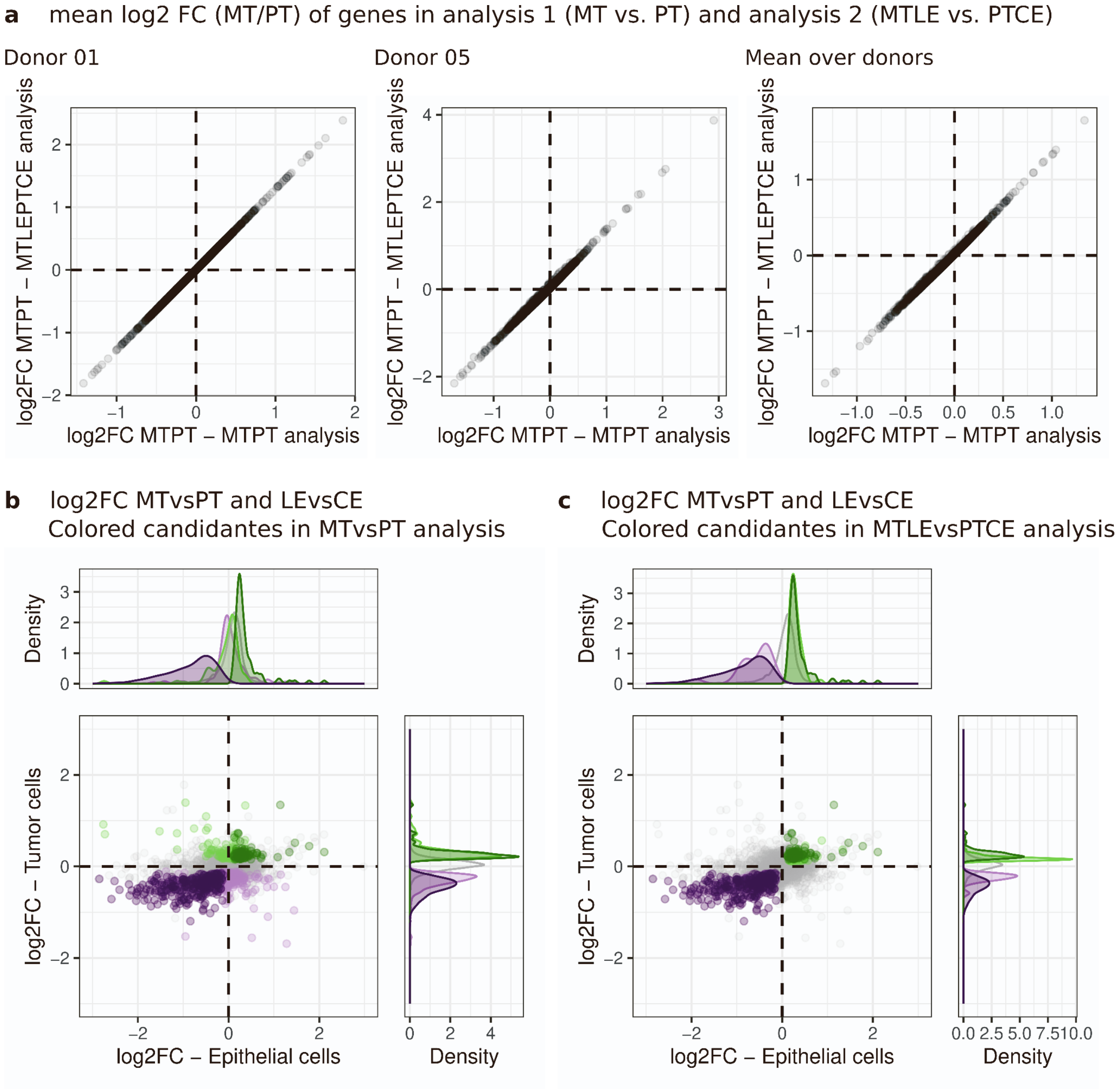

Figure S4: Differences in analysis and candidates between MT vs PT and MTLE vs PTCE analysis. a) *Log2 FC of primary tumor vs metastasis comparison (Figure 2) and primary tumor vs metastasis & liver vs colon epithelial cells comparison (Figure 3) plotted against each other in donor 1 (left), donor 5 (middle) and mean over all donors (right).* **b)** *Mean log2 fold change of metastasis vs primary tumor plotted against the mean log2 fold change of liver vs colon epithelial cells estimated over the donors. Genes, which are defined as both, cancer specific (Figure 2b) and cancer & surrounding tissue specific (Figure 3c), are depicted in dark purple (primary tumor & primary tumor + colon epithelial specific) and dark green (metastasis & metastasis + liver epithelial specific), while genes which are only cancer specific defined by the metastasis vs primary tumor analysis (Figure 2b) are light purple (primary tumor) and light green (metastasis). Genes with no specificity are gray. (Four outlier genes are not visualized in the plot.)* **c)** *As b) but light colored genes are genes which are only cancer & surrounding tissue specific (Figure 3c, light purple = primary tumor & colon epithelial, light green = metastasis & liver epithelial).*

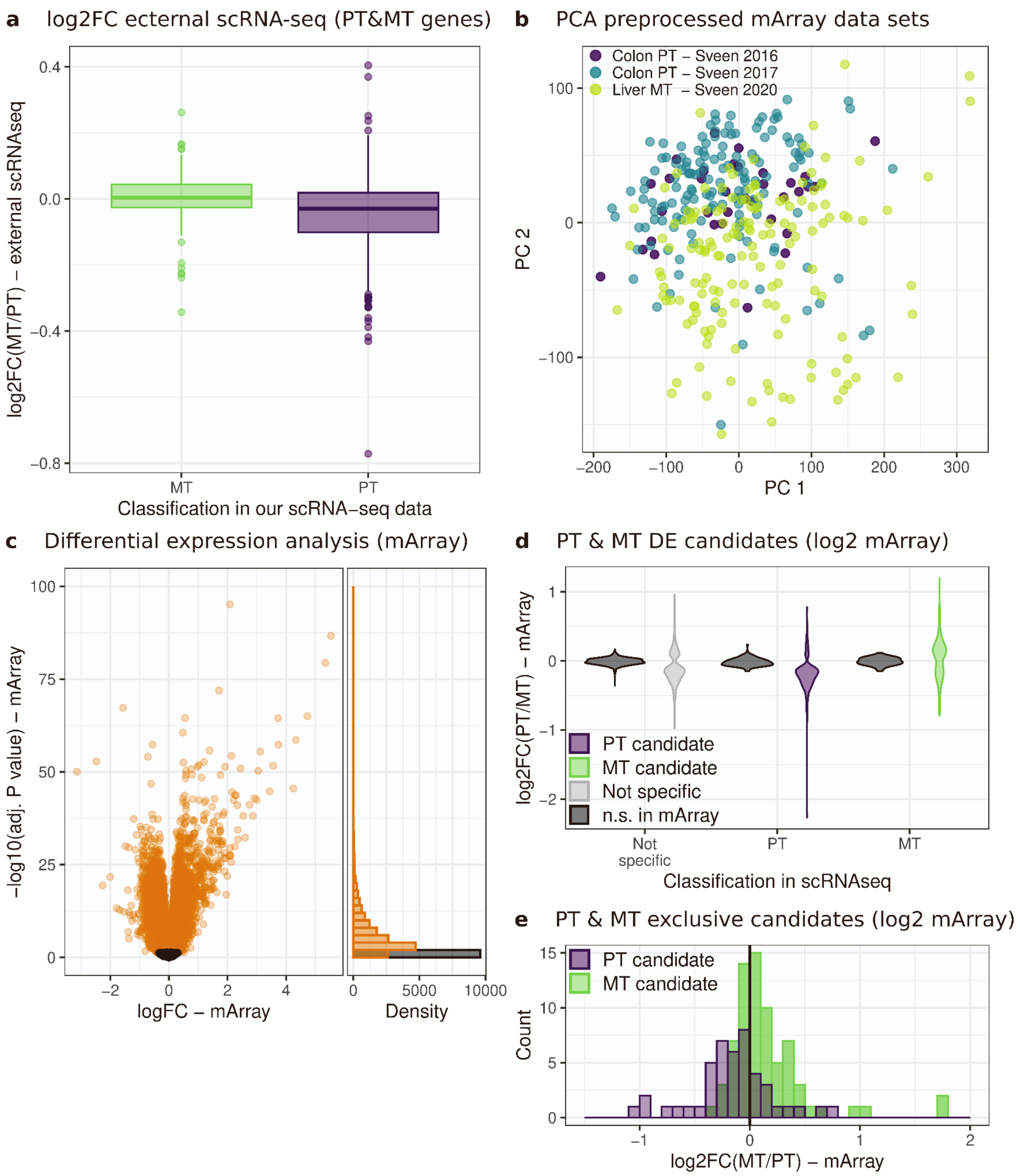

Figure S5: Validation of tissue specific candidate genes with external data sets. a) *Log2 FC from metastasis to primary tumor cells in external scRNA-seq data plotted by their classification in our data (PTCE candidates = purple, MTLE candidates = green). Box limits represent the interquartile range (IQR; 25th to 75th percentiles), the solid line inside the box indicates the median and the whiskers extend to data points within (1.5 x IQR) from the quartiles.* ***b)*** *PCA of preprocessed microarray data sets, colored by dataset and sample origin.* ***c)*** *Log2 FC and adjusted p-values of external microarray data from MT compared to PT. Genes that are differentially expressed (adjusted p-value =< 0.05) are depicted in orange, all others in black.* ***d)*** *Log2 FC from MT to PT samples of external microarray data set plotted by their classification in our data (PT specific = purple, MT specific = green, non-specific = gray). Genes which are not differentially expressed in the microarray dataset (adjusted p-value < 0.05) are colored in black.* ***e)*** *Log2 FC from MT to PT samples of external microarray data set of all genes which have been found to have a tissue exclusive expression in our data (PT exclusive = purple, MT exclusive = green).*

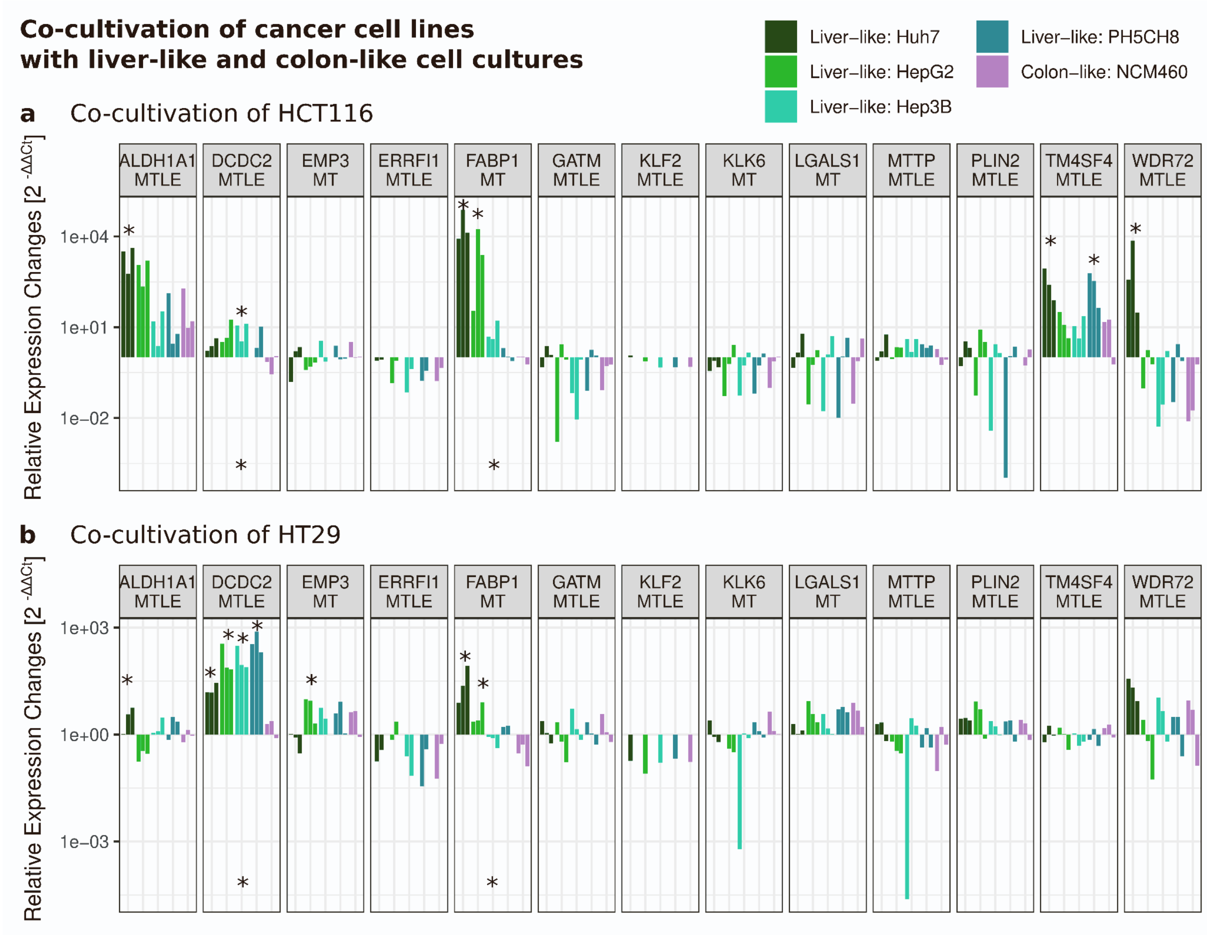

Figure S6: Co-cultivation of cancer cell lines with liver or colon epithelial derived cell cultures. a) *Co-cultivation of HCT116 cells with four liver-like cell lines (dark green, light green, turquoise, blue) and one colon-like cell line (violet).* ***b)*** *Co-cultivation of HT29 cells. Colors as in a. Data of three independent experiments are shown. For each experiment, the average of three technical replicates is shown. Two-tailed t-test were applied to test for differences between every liver-like cell line vs the colon-like cell line individually (* over the bars p-value<0.05) and between all liver-like cell lines vs the colon-like cell line (* under the bars p-value<0.05).*

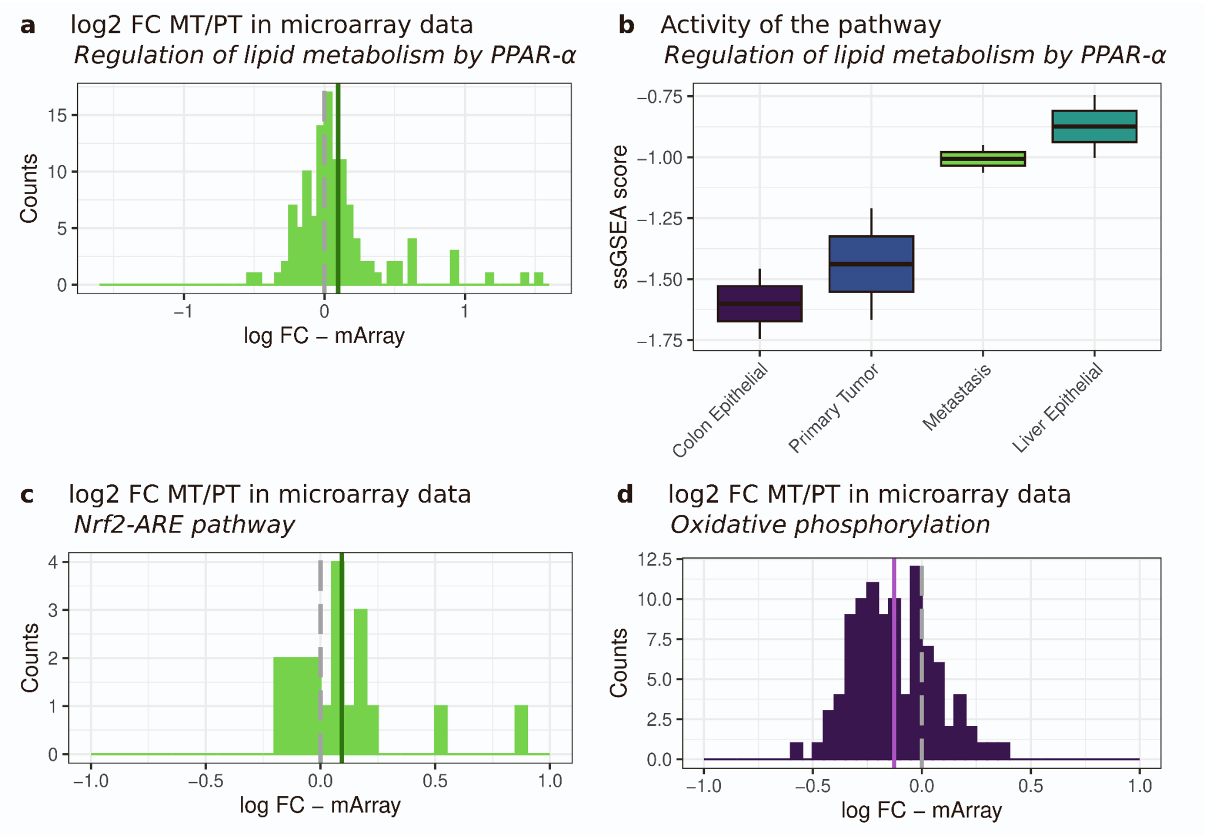

Figure S7: Pathway analysis. a) *Log2 fold changes in between the primary tumor and metastasis cells (MT/PT) in external microarray data for all genes involved in the KEGG pathway 'Regulation of lipid metabolism by PPAR-α', which is higher expressed in the metastasis and liver epithelial cells of our scRNA-seq data. The mean gene expression of all pathway genes in the external microarray data is visualized with a dark green line.* ***b)*** *Tissue-specific pathway activity based on the log2 pseudobulk donor specific expression levels of the KEGG pathway 'Regulation of lipid metabolism by PPAR-α'. Box limits represent the interquartile range (IQR; 25th to 75th percentiles), the solid line inside the box indicates the median and the whiskers extend to data points within (1.5 x IQR) from the quartiles.* ***c)*** *As in a) but for the REACTOME pathway 'Nrf2-ARE pathway', which is higher expressed in the metastasis and liver epithelial cells of our scRNA-seq data. The mean gene expression of all pathway genes in the external microarray data is visualized with a dark green line.* ***d)*** *As in a) but for the KEGG pathway 'Oxidative phosphorylation', which is higher expressed in the primary tumor and colon epithelial cells of our scRNA-seq data. The mean gene expression of all pathway genes in the external microarray data is visualized with a light purple line.*
